# Underestimation of Future Agricultural Soil N_2_O Emissions and Abatement Needs

**DOI:** 10.1111/gcb.70919

**Published:** 2026-05-15

**Authors:** Chaoqun Lu, Linchao Li, Wilfried Winiwarter, Josep G. Canadell, Weihang Liu, Hanqin Tian

**Affiliations:** ^1^ Department of Soil and Environmental Sciences University of Wisconsin‐Madison Madison Wisconsin USA; ^2^ Department of Ecology, Evolution, and Organismal Biology Iowa State University Ames Iowa USA; ^3^ International Institute for Applied Systems Analysis Laxenburg Austria; ^4^ Institute of Environmental Engineering University of Zielona Góra Zielona Góra Poland; ^5^ CSIRO Environment Canberra Australian Capital Territory Australia; ^6^ Center for Earth System Science and Global Sustainability Boston College Chestnut Hill Massachusetts USA; ^7^ Department of Earth and Environmental Sciences Boston College Chestnut Hill Massachusetts USA

**Keywords:** agricultural soil N_2_O emissions, climate change, dynamic vs. static emission factors, future projection, nitrogen regulation policy scenarios

## Abstract

Agricultural soils are the largest human‐induced source of nitrous oxide (N_2_O) due to the extensive fertilizer use in crop production. Despite progress made in global N_2_O budget accounting, using the IPCC‐derived static emission factors (EF) limits our capability to project future changes in agricultural N_2_O emissions and identify cost‐effective mitigation strategies. Here, we use a physics‐informed AI‐driven dynamic EF modeling framework to project direct agricultural soil N_2_O emissions induced by mineral fertilizer additions under various climate and nitrogen (N) regulation policy scenarios. Compared to projections based on static EF, our study yields higher future N_2_O emissions by 0.3–1.1 Tg N year^−1^ across scenarios by 2050, implying greater abatement needs to combat climate change. The gap between static and dynamic EF approaches is projected to widen to 29%–34% by mid‐century. Under moderate‐ to high‐ambition N regulation policies, we project that a 25% emission reduction can be reached before 2050, while a 45% reduction is only attainable under high‐ambition N policies and strong climate action. The potential for N_2_O mitigation due to a policy shift varies substantially among regions, with seven top source regions contributing 76%–87% of global N_2_O reduction. Adopting N_2_O reduction technologies in hotspot areas would significantly accelerate the timeline for achieving the 25% reduction goal. Improving fertilizer management on croplands can provide climate benefits comparable to, or even exceeding, those of enhancing soil carbon sequestration, particularly in regions with low nitrogen use efficiency. Our findings highlight the higher mitigation potential of targeting N_2_O emission hotpots and the urgency of implementing policy shifts.

## Graphical abstract

Agricultural soils are a major source of rising atmospheric N_2_O concentrations, yet future emission projections remain highly uncertain. Most assessments rely on static emission factors (EFs) that assume constant N_2_O release per unit of fertilizer, overlooking how climate change and evolving fertilizer practices may alter these factors. Using a physics‐informed, AI‐driven dynamic EF framework, we project future agricultural soil N_2_O emissions and regional mitigation potential across multiple climate and fertilizer policy scenarios. We find that static EF approaches substantially underestimate future emissions and abatement needs. Our results demonstrate that achieving a 45% reduction goal in agricultural soil N_2_O emissions by 2050 will require high‐ambition nitrogen regulation policies, strong climate action, and region‐specific strategies, highlighting the inadequacy of uniform global approaches to agricultural mitigation.

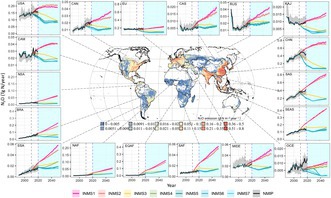

## 1 Introduction

Increasing attention has been paid to exploring the earth's potential to reduce greenhouse gas (GHG) emissions to net zero for controlling global warming (Griffis et al. 2017; Hergoualc'h et al. 2019; Tian et al. 2024). While anthropogenic carbon dioxide emissions have become relatively stable in recent decades (Friedlingstein et al. 2023), the atmospheric concentration of nitrous oxide (N_2_O) is growing more rapidly than at any time in the historical observation records (Li et al. 2024). Among all the source sectors, global human‐induced N_2_O emissions have increased by 40% since 1980, and over half of this increase is attributed to the rise of direct emissions from agricultural soils (Tian et al. 2024, 2020). Since the invention of the Haber‐Bosch process, synthetic nitrogen (N) fertilizer use has produced food for about half of the world's population (Erisman et al. 2008). However, this has inevitably increased N_2_O emissions into the atmosphere, which are projected to continue rising (Syakila and Kroeze 2011).

To limit global warming to no more than 1.5°C, it is agreed that global GHG emissions need to be cut by 45% from the 2010 levels by 2030 and reach net zero by 2050, and constraining warming to 2°C requires a 25% reduction by 2030 (Masson‐Delmotte et al. 2018; UNEP 2024). Achieving the net zero target demands an improved understanding of both the mitigation potential and emission gap in agricultural soils, which remain a major source of “hard‐to‐abate” residual emissions (Edelenbosch et al. 2024; Lecocq 2022). Recently, a wide variety of studies have investigated the policy and technology opportunities to reduce global anthropogenic emissions of N_2_O (Kanter, Winiwarter, et al. 2020; Winiwarter et al. 2018). Yet, our capacity to manage and mitigate soil N_2_O emissions is critically hampered by the extreme spatiotemporal heterogeneity in these emissions (Butterbach‐Bahl et al. 2013; Lawrence et al. 2021) and the physical, technical, and resolution limitations of current assessment methods (i.e., mostly based on IPCC guidelines for GHG emission inventories) (Hergoualc'h et al. 2019). For instance, the IPCC guidelines use a fixed “emission factor‐EF” approach to estimate N_2_O emissions per unit of fertilizer input. However, this widely applied method ignores the impacts of spatially heterogeneous and temporally dynamic environmental factors such as soil properties, crop‐specific management practices and climate variability. Although empirical and machine learning‐based approaches were used in developing quantitative insights into N_2_O mitigation potential, the non‐linear dynamics of EF in response to climate change are not adequately addressed, as the measurement data used for model training often span only a few years (Cui et al. 2024, 2021; Li et al. 2024). These limitations make it hard to identify the most cost‐effective mitigation strategies to reduce N_2_O emissions while meeting the growing food demands of the global population.

Here, we employed a newly developed AI‐empowered modeling framework, Dym‐EF (Li et al. 2024), to project direct soil N_2_O emissions associated with N fertilizer inputs in croplands and pasture under various climate and N regulation policy scenarios. Different from the static EF approaches used in IPCC guidelines, the Dym‐EF integrates machine learning approaches and eight process‐based models assembled for the global N_2_O Model Intercomparison Project‐Phase 2 (NMIP2) (Tian et al. 2024) to capture the non‐linear responses of direct N_2_O EF to multiple environmental factors such as climate, soil properties, and management practices.

Although our previous work (Li et al. 2024) projected future increases in EF, this study advances those findings by demonstrating substantial regional variability in N_2_O mitigation potential across fertilizer input levels and by revealing marked divergence in projected N_2_O emissions when dynamic and static EF approaches are applied. More specifically, first, we quantify the future changes in global direct N_2_O emissions induced by synthetic N fertilizer addition under seven scenarios by comparing them with the 2010 baseline and the 2011–2020 emissions under current policies; we then project the mitigation potentials by assessing the difference in the emissions of 2050 between best‐case and business‐as‐usual scenarios. Second, we identify hotspot areas with high emission rates and top source regions/countries with a large share of the global total. Third, we investigate the role of “low‐hanging fruits” by assuming that hotspot areas have a high priority to adopt N_2_O reduction technologies such as nitrification inhibitors on top of each scenario. This study provides quantitative insights on dynamic and static EF approaches that differ or agree on the future N_2_O emission change trajectories and mitigation potentials over space and time, identifying more robust projections toward the stated emission reduction targets of 25% and 45%.

We follow the scenario set developed by the project “Toward an International Nitrogen Management System (INMS)” (Kanter, Winiwarter, et al. 2020) that combines future changes in climate (SSP scenarios) and N policy interventions with high, moderate, and low ambition levels within the framework of Shared Socioeconomic Pathways (SSPs, see *Methods*). For example, the business‐as‐usual (BAU, termed INMS1 here) and low‐ambition (INMS2) scenarios combine BAU or low‐ambition N policies with SSP5‐8.5 and SSP2‐4.5 climate data, respectively. Moderate to high‐ambition N regulation policies, in medium (INMS3), high (INMS4), best‐case (INMS5), and best‐case plus scenarios (INMS6), are paired with SSP2‐4.5. The bioenergy scenario (INMS7) integrates high‐ambition N regulation with strong climate action (SSP1‐2.6). These N policy scenarios define different timelines for nitrogen use efficiency (NUE) improvement at a country and regional level, which drives fertilizer consumption patterns from 2010 to 2050. While the analysis is based on the INMS framework, the differences in N_2_O emissions reported here do not account for altered N_2_O emissions associated with changes in diet structure, food waste reduction, or manure management, except as much as these factors indirectly affect fertilizer application rates.

## 2 Methods

### 2.1 Dynamic vs. Static EF Modeling Approach

The IPCC‐derived empirical N_2_O estimation approach, quantifying how much anthropogenic N input ends up as N_2_O released into the atmosphere according to static emission factors (EF), has been widely used for global N_2_O assessment and integrated cost–benefit assessment models (Hergoualc'h et al. 2019). This is partially because the anthropogenic N inputs within specific land use types can be aggregated and easily accessed at the country level. However, this methodology adopts a fixed EF value and ignores the impacts of spatially heterogeneous and temporally dynamic crop‐specific management practices (e.g., fertilizer management, irrigation, tillage, and tile drainage) and climate variability (Shcherbak et al. 2014), leading to biases in N_2_O estimations. Despite much progress made in global N_2_O budget accounting, we do not have robust geospatial projections of N_2_O emissions yet, especially for agricultural sectors, considering EF's non‐linear responses to climate changes, soil properties, and various levels of N inputs (Li et al. 2024), see the section of “Environmental and management factors regulating N_2_O EF dynamics” in Supporting Information for more detailed information.

In contrast, the process‐based modeling approach aims at providing better estimates by simulating the non‐linear responses of N_2_O emission to changes in climate, soil properties, land use, and management practices. They are obviously more advantageous than the static EF approach in quantifying biogenic N_2_O emission rates in various ecosystems and attributing the spatiotemporal variations in N_2_O emissions to different driving forces (Lu et al. 2022; Xu et al. 2012). However, process‐based modeling is knowledge‐intensive, time‐consuming in data and model preparation, and computationally expensive. All of these make it less practical to inform the cost‐effective N_2_O mitigation assessment and policy identification. Additionally, significant variations in soil N_2_O emission estimates arise from differences in how biogeochemical processes are represented across models and various sources of input data used to drive the models (Li et al. 1996; Xu et al. 2012).

To provide an accurate historical assessment, the N_2_O Model Intercomparison Project (NMIP) was launched to develop a multi‐model ensemble estimate on the budget of global/regional soil N_2_O emissions using consistent driver datasets and simulation protocols (Tian et al. 2024, 2018, 2019). The most recent NMIP phase‐2 activity has updated the global biogenic N_2_O emission estimates to 2021, which provides a century‐long database at a monthly time step and a resolution of 0.5° by 0.5°. The NMIP2 project includes eight state‐of‐the‐art process‐based Terrestrial Biosphere Models (TBMs) for estimating N_2_O emissions from natural and agricultural soils during 1980–2020. These models consider nitrogen inputs from N deposition, biological fixation, and N fertilizer use, although some do not account for manure‐related processes. In this study, we used the multi‐model median from NMIP2 to represent state‐of‐the‐art N_2_O emission estimates at a half‐degree resolution. The consistency in NMIP2 input data, simulation protocol, and variables of interest lays a solid foundation for the Dynamic EF approach used in this work. More detailed information about NMIP2 can be found shown in (Li et al. 2024). By comparing the projections of agricultural soil N_2_O emission between static and dynamic EF approaches, our study aims to improve the projection robustness in N_2_O emissions and mitigation potentials under various climate and policy scenarios across the globe.

#### 2.1.1 Dynamic EF Approach

Estimating N_2_O emissions with process‐based models requires substantial computational resources, especially when factoring in various nitrogen management practices, which significantly increase the computational load. To overcome this challenge, we adopted a hybrid modeling framework, Dym‐EF, to project the annual time‐series N_2_O emissions from agricultural soils at a resolution of 0.5° by 0.5° under various climate and N policy scenarios during 2010–2050. The Dym‐EF integrates machine learning with eight process‐based N_2_O models (including CLASSIC, DLEM, ELM, ISAM, LPX‐Bern, OCN, ORCHIDEE, and VISIT) to dynamically learn the relationship between N_2_O emission factors (EFs) and multi‐source environmental data during 1960–2020. This method captures the dynamic nature of EFs for N_2_O emission simulations and has been thoroughly validated. It is proven that the Dym‐EF is able to reproduce the spatial and temporal dynamics of EF, as estimated by the NMIP2 model ensembles. In this study, we further evaluate the simulated EF by comparing it with EF observations from multiple sites across the global croplands (Figure S1). It shows that the Dym‐EF can well capture the variations of N_2_O EF values across the aridity and SOC gradient with an RMSE of 0.3% and 0.2%, respectively (Figures S2 and S3). Generally, the Dym‐EF model demonstrates strong performance in reproducing median estimates of annual N_2_O emissions associated with fertilizer use from the NMIP2 multi‐model ensemble (Figure S4). The Dym‐EF model was then used to project direct soil N_2_O EF under seven future scenarios, which encompass a range of climate and nitrogen regulation policies at different ambition levels (Li et al. 2024). Due to the structure of the INMS scenarios (Table S1), future projections begin in 2010, overlapping by ten years with the historical N_2_O assessment. This overlap allows for a comparison between the estimates of N_2_O emissions under current policies and various N policy scenarios at low, moderate, and high ambition levels, each paired with corresponding weak and strong climate action. Future input data used in the simulations include climate, land use, and nitrogen fertilizer inputs. The spatial patterns of cropland and pasture distribution follow the gridded land‐use datasets used in the NMIP2 framework at 0.5° resolution. Historical gridded fertilizer application data provide the baseline spatial distribution of nitrogen inputs. Future fertilizer inputs are generated by scaling the grid‐level baseline using the country‐ or region‐level fertilizer trajectories defined in the scenario framework. This approach ensures that total fertilizer consumption follows the scenario pathways while preserving the baseline spatial heterogeneity of fertilizer application across grid cells.

The multi‐source environmental data that have been used to train the Dym‐EF over the historical period and drive the model projections include time‐series data of climate variables (monthly precipitation, temperature, and yearly aridity index), agricultural management practices (e.g., irrigation), and time‐invariant data such as soil properties (pH, initial soil organic carbon content, soil bulk density, sand content, and clay content). Historical climate data were sourced from the Climate Research Unit (CRU) database (https://data.ceda.ac.uk/badc/cru/data/). Future climate data were obtained from the CMIP6 database including 37 Global Climate Models (GCMs) (https://esgf‐node.llnl.gov/projects/cmip6/). Soil data were obtained from the Harmonized World Soil Database v1.2. Historical nitrogen input data were derived from the NMIP2 model input dataset (Tian et al. 2022). Annual time‐series cropland and pasture area data were sourced from the HYDE 3.2 database (ftp://ftp.pbl.nl/hyde). All these data sets are processed to be consistent at the same half‐degree resolution.

#### 2.1.2 Static EF Approach

In this study, we selected the GAINS (Greenhouse Gas—Air Pollution Interactions and Synergies) model as an example of the static EF approach to compare with our estimations using dynamic EF framework. The GAINS model provides a comprehensive assessment framework for considering N_2_O emissions from various factors and source sectors, particularly for future scenarios under different assumptions. However, GAINS relies on fixed EF (i.e., IPCC Tier‐1 EF value of 1% in this study) to estimate direct agricultural soil N_2_O emissions, without considering non‐linear EF responses to climate change, variations in cropping system and soil properties, or N input levels along with diversified agricultural management practices. The GAINS model offers N_2_O emission projections in 172 regions at 5‐year intervals from 1990 to 2050, incorporating factors like energy consumption, agricultural production, population growth, and industrial activities (Amann et al. 2011). The baseline scenarios were based on FAO data representing current fertilizer consumption levels. Reductions due to opportunities of reducing mineral fertilizer additions are implemented in the scenarios, further emission reductions implemented in GAINS by the addition of nitrification inhibitors have been reserved for the evaluation of hotspots.

### 2.2 Scenario Data for N_2_O Emission Projections

The INMS scenarios provide a comprehensive framework that considers major reactive N sectors and addresses related social and environmental issues, such as food security and climate change. Recognizing the spatial heterogeneity of N imbalance across the world, the INMS scenarios set up different timelines for OECD/non‐OECD countries with high/medium or low N use to reach the national‐level NUE targets (Zhang et al. 2015). They include two low‐ambition scenarios (Business‐as‐usual and Low N regulation, INMS1‐2), one moderate‐ambition scenario (INMS3), and four high‐ambition scenarios (High N regulation, Best‐case, Best‐case Plus, and Bioenergy, which are INMS 4–7). The scenarios reflect varying levels of improvement in NUE that were developed to meet the SDG targets. The high‐ and moderate‐ambition N policies represent contrasting fertilizer input levels. These policies envision different timelines for meeting national‐level NUE targets before 2050 through best management practices or improvements in fertilizer technology. In contrast, the low‐ambition policy scenario assumes that NUE targets will not be met, leading to sustained high levels of fertilizer inputs or further increases to support the growing population. For instance, in OECD countries, the high and moderate‐ambition policy scenarios assume the national‐level NUE target can be achieved by 2030 and 2050, respectively, while no targets are met under the low‐ambition policy scenario. For non‐OECD countries, depending on each country's economic and N use status, the scenarios outline different trajectories for NUE changes, defining whether and when the national‐level targets are reached (Table S1). This, in turn, projects nitrogen fertilizer consumption in each region or country from 2010 to 2050 (Kanter, Winiwarter, et al. 2020). Among them, the “best‐case” scenario reflects ambitious climate action combined with sustainable agriculture and low‐meat dietary transitions under SSP1. The best‐case plus scenario further strengthens these ambitions by incorporating substantial dietary shifts and reductions in food loss, while the bioenergy scenario highlights improved bioenergy production as critical for meeting the 1.5°C and 2°C climate targets (Kanter, Chodos, et al. 2020; Kanter, Winiwarter, et al. 2020; Li et al. 2024). We used the annual changes in region/country‐level fertilizer consumptions from each INMS scenario to develop future N fertilizer input data for the Dym‐EF simulations, with the half‐degree historical database of global fertilizer management serving as a baseline. Specifically, the region‐ or country‐level fertilizer trajectories were not applied uniformly across all grid cells; instead, they were used to scale the historical gridded fertilizer inputs, so that total fertilizer use followed the scenario pathway while the baseline spatial pattern at 0.5° resolution was retained. This approach links country‐ or region‐level scenario trajectories with the gridded Dym‐EF simulations while preserving the spatial heterogeneity represented in the baseline fertilizer dataset. The historical gridded database of N fertilizer uses (Tian et al. 2022) is also a critical input dataset for NMIP2 modeling assessment and historical training data for the Dym‐EF model. The region/country‐level fertilizer consumption data were used in the GAINS simulation and they are slightly different from the gridded database (Figure S5).

This study uses future climate data from 37 Global climate models (GCMs) under three SSP scenarios (SSP126, SSP245, and SSP585) to match the N regulation policy scenarios as defined in the INMS project (Kanter, Winiwarter, et al. 2020). Specifically, the projected N fertilizer data under the BAU and low‐ambition N policy scenarios, combined with SSP5‐8.5 and SSP2‐4.5 climate data (representing low and moderate climate action), respectively, are used to drive the Dym‐EF model to project N_2_O emissions for BAU (INMS1) and low ambition (INMS2). In addition, the SSP2‐4.5 climate data are adopted in moderate to high‐ambition N policy scenarios, including medium N regulation (INMS3), high N regulation (INMS4), best‐case (INMS5), and best‐case “plus” (INMS6). The bioenergy scenario (INMS7) combines high‐ambition N regulation policy and strong climate action (SSP1‐2.6). It is noteworthy that, although we follow the INMS framework, the scenarios we examined here only reflect a combined impact of climate and N fertilizer use scenarios under different ambition‐level policies. To harmonize historical and future climate data, we applied a delta approach to bias‐correct the climate data, as follows:

(1) $${\mathrm{GCM}}_{\text{bias}}={\mathrm{GCM}}_{\text{project}}-\left({\mathrm{GCM}}_{\mathrm{His}}-\mathrm{OBS}\right)$$

where the GCM_bias_ is the GCM after bias correction, GCM_project_ is the future projections of GCMs, the GCM_His_ is the historical GCM data, and OBS is the reanalysis data of climate observations. The reanalysis data were collected from the Centre for Environmental Data Analysis (CEDA) Archive. We resampled the climate data from 37 GCMs to the resolution of 0.5° × 0.5°.

### 2.3 Projecting N_2_O Emissions and Mitigation Potential of Adopting N_2_O Reduction Technology in Hotspot Areas

Using this hybrid modeling framework, we projected direct soil N_2_O emissions associated with N fertilizer use in croplands and pasture across the globe at a resolution of 0.5° × 0.5° during 2010–2050 under seven scenarios. To limit global warming to no more than 1.5°C, global GHG emissions need to be reduced by ~45% (compared to the 2010 levels) by 2030 and reach net zero by 2050 (Gladilshchikova et al. 2018; UNEP 2024). A 25% reduction goal is set to limit the global temperature increase to 2.0°C above the pre‐industrial levels. Despite the widely reported goals of GHG reduction to fulfill the Paris Agreement, there is no specific allocation for individual gases. Therefore, in this study, we adopt the general GHG reduction goal to assess the extent of agricultural soil N_2_O mitigation potential. These reduction levels are used here only as system‐wide mitigation benchmarks to contextualize the scale of agricultural N_2_O mitigation needs, rather than as gas‐specific mitigation targets. In addition, we contextualize state‐of‐the‐art estimates of agricultural N_2_O emissions from the recent decade (2011–2020, estimated by both NMIP2 and Dym‐EF) by comparing them to N_2_O projections under seven combinations of climate and N‐relevant SSP scenarios starting from 2010. Given the long lifetime of N_2_O in the atmosphere, we also examined the cumulative N_2_O emissions associated with fertilizer use under different climate and policy scenarios, and the timeline when the reduction target could be achieved under each scenario by comparing the cumulative N_2_O emission with the sum of N_2_O at the 2010 level.

In this study, we define hotspots by first calculating the annual N_2_O emission rate (g N m^−2^ year^−1^) for each 0.5° × 0.5° grid cell and then ranking all cells in descending order. We label the top portion of that ranked list (for instance, the top 10% of grid cells with highest N_2_O emission rate) as the hotspot region. This threshold‐based approach highlights the areas emitting disproportionately high levels of N_2_O, enabling more targeted analysis and more efficient prioritization of mitigation efforts.

The use of nitrification inhibitors can further enhance the potential for N_2_O mitigation. These inhibitors suppress soil microbial activity, reducing emissions by 34%–38%, as demonstrated experimentally (Fan et al. 2022; Recio et al. 2020; Winiwarter et al. 2018). In this study, following the effectiveness parameter values set in the GAINS model, we assume that nitrification inhibitors can suppress microbial activity and reduce N_2_O emissions by 34%, without reducing fertilizer input or fertilizer cost (Winiwarter et al. 2018). Given that these inhibitors are more effective in areas with high N_2_O emissions, we quantified the additional N_2_O mitigation potential by expanding the NI adoption area from the grid cells with highest N_2_O emission rate to the lowest. Under each scenario, we examined how the timeline to achieve N_2_O reduction goal could be expedited and identified the least NI adoption area to meet the 25% and 45% reduction goal by prioritizing hotspot grid cells.

### 2.4 Uncertainty Assessment

The projection uncertainty range for each individual scenario is quantified by the estimation range from the 25th to 75th percentiles driven by future climate data from the 37 GCMs. The annual median estimates from 37 simulations are used to represent N_2_O emissions projected under each scenario. In addition, we use the min‐max range of N_2_O projections to demonstrate the N_2_O changes and mitigation potential between scenarios (e.g., policy shifted from BAU/low ambition level to high ambition levels). The Dym‐EF approach used in this study aims to reproduce the median EF estimated by eight process‐based models that participate in the historical N_2_O assessment of NMIP2 (Tian et al. 2024). Although these models used consistent input data and simulation protocols, large uncertainties remain in estimating N_2_O emissions due to model structure and parameter values (O'Sullivan et al. 2022; Vogel et al. 2024). While we acknowledge the uncertainties arising from cross‐model variations, which are a common feature of model intercomparison projects, we adopt the ensemble median as the best available, state‐of‐the‐art estimates of agricultural soil N_2_O emissions and use them to train the Dym‐EF framework. This choice reflects our emphasis on characterizing robust central tendencies in EF dynamics, rather than attributing differences to individual model structures or parameterizations. Consequently, a detailed analysis of cross‐model discrepancies is beyond the scope of this study, particularly given that inter‐model divergence can be substantial and spatially heterogeneous at the global scale.

## 3 Results and Discussion

### 3.1 Future Projections of Global Agricultural Soil N_2_O Emissions

**FIGURE 1:**
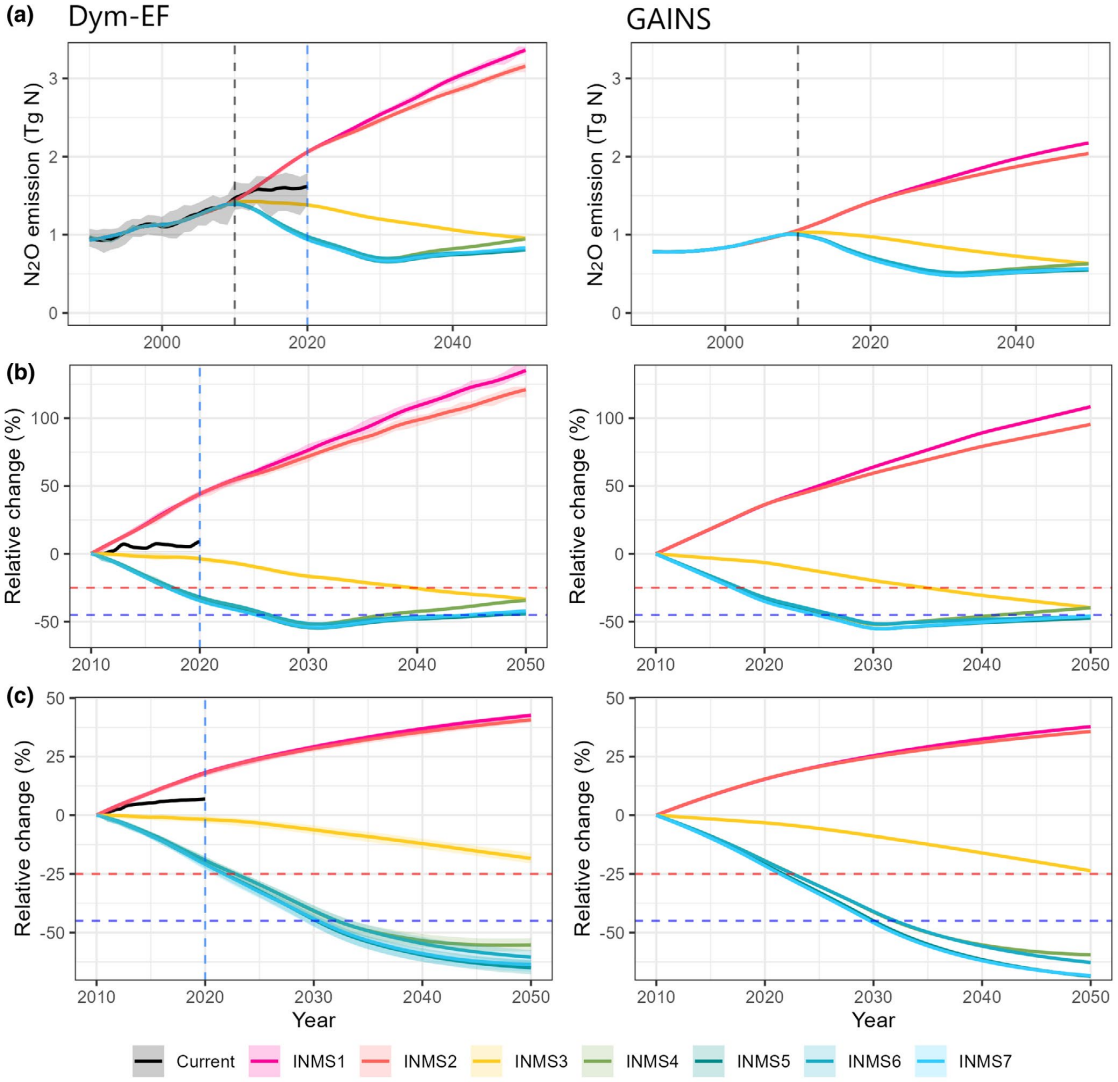
Global cropland and pasture N_2_O emission changes under different scenarios towards 2050. The time series of N_2_O emissions from 1990 to 2050 under seven different scenarios. (b) The relative change in N_2_O emissions compared to the year 2010. (c) The relative change in cumulative N_2_O emissions compared to 2010. The black line represents the N_2_O emissions projected by the dynamic EF that reproduce the median values of the NMIP2 model ensemble (min‐max shown as the gray shaded area). The shaded area for INMS1‐7 represents the estimation range from the 25th to 75th percentiles driven by climate data from the 37 GCMs. The black dashed vertical line marks the year 2010, and the blue dashed vertical line indicates 2020. The red horizontal lines represent the 25% reduction, and the blue horizontal line for the 45% reduction relative to the 2010 baseline. The scenarios include INMS1 (Business as usual—low ambition), INMS2 (Low N regulation—low ambition), INMS3 (Medium N regulation—moderate ambition), INMS4 (High N regulation—high ambition), INMS5 (Best‐case—high ambition), INMS6 (Best‐case plus—high ambition), and INMS7 (Bioenergy—high ambition). Static‐EF stands for the estimates of N_2_O emissions based on IPCC tier‐1 EF (1%), and Dym‐EF for N_2_O emission estimates based on dynamic EFs.

Different policy ambition levels delineate a wide range of scenarios in nitrogen inventions that could affect food security and climate mitigation potentials (Kanter, Winiwarter, et al. 2020; Winiwarter et al. 2018). By incorporating the SSP climate scenarios and N regulation policies with different ambition levels into the well‐validated Dym‐EF model, our projections demonstrate contrasting trajectories of agricultural N_2_O emission changes across the scenarios. Under current policies, we find that N_2_O emissions from fertilized agricultural soils have increased by 11% during the past decade. While this increase has not followed the trend of the worst‐case scenario, it falls between the projected trajectories with policies at low and moderate ambition levels (black line in Figure 1). Under the BAU and low ambition N regulation scenarios, combined with SSP5‐8.5 and SSP2‐4.5 climate data (representing low and moderate climate action), respectively, agricultural soil N_2_O emissions are projected to rise to 2.42–2.59 Tg N year^−1^ (min‐max range across scenarios and climate inputs) by 2030 and to 3.07–3.28 Tg N year^−1^ by 2050 (Figure 1). These values represent an increase of 67%–81% by 2030 and 115%–138% by 2050 compared to the 2010 levels of 1.41–1.46 Tg N year^−1^. However, under the combination of high‐ambition N regulation policies and moderate‐ to strong‐climate action (i.e., SSP1‐2.6 and SSP2‐4.5), N_2_O emissions could decrease to 0.64–0.71 Tg N year^−1^ by 2030 (a reduction of 51%–56%) and 0.81–0.95 Tg N year^−1^ by 2050 (a reduction of 32%–45%). By contrast, static‐EF approaches, such as the GAINS model in this case, leads to a lower estimate of agricultural N_2_O emissions in 2010 by 26%–30% (1.04 Tg N year^−1^ from GAINS vs. 1.41–1.46 Tg N year^−1^ from NMIP2 and Dym‐EF) (Tian et al. 2024). A similar difference was reported in earlier publications. For instance, the direct agricultural soil N_2_O emissions estimated by EDGAR and FAO was 23% lower than that from NMIP2 (2.0 Tg N year^−1^ vs. 2.6 Tg N year^−1^ associated with chemical N fertilizer and manure N applications in 2020, Tian et al. 2024). Furthermore, our results suggest that the discrepancy between static and dynamic EF approaches will be widen by 2050, reaching 29%–34% across scenarios. Specifically, projections indicate that N_2_O emissions estimated using static EFs will be 0.3–1.1 Tg N year^−1^ lower than those derived from the dynamic EF approach in 2050, depending on the scenario considered (Figure 1). In addition, we find that the static EF‐based future projections result in overly optimistic estimates when compared with ours. For example, compared with their estimate of 2010 baseline, the static‐EF approach projects a decline of 40%–46% in N_2_O emissions by 2050 under the combination of high‐ambition N regulation policies and medium to strong climate action (SSP1‐2.6 and SSP2‐4.5), a reduction of 40% under moderate ambition scenarios, and an increase of 95%–108% under BAU and low ambition scenarios (Figure 1a,b). This is primarily because the extensively used static EF approach does not account for EF increase in response to enhanced N input levels and climate warming, or reductions in EF with lower N inputs and cooling climate (Li et al. 2024). For instance, the global average EF for agricultural soils is estimated at 1.18%–1.22% in 2010, exceeding the IPCC Tier‐1 EF value of 1%. This EF is further projected to increase by 12%–17% and 4%–11% by 2050 under low‐ and high‐ambition N regulation policy scenarios, respectively, when combined with weak versus strong climate mitigation actions (Li et al. 2024). Recognizing the positive feedback loop between climate warming and N_2_O emission growth is important, particularly if limited or no climate action are taken. Increasing evidence shows that a warming climate can exacerbate N_2_O emissions by increasing emission factors (Harris et al. 2019; Li et al. 2024).

Our results also highlight the urgency of implementing strong climate action to reduce agricultural N_2_O emissions under existing nitrogen management policies. Under the BAU and low‐ambition N policies, global agriculture N_2_O emission in 2050 would double or more than double the 2010 level. Both Dym‐EF and static EF approaches agree that if the high‐ambition N regulation policies had been implemented since 2010, under the SSP1‐2.6 and SSP2‐4.5 climate scenarios, the 25% reduction goal would have been reached by 2018, and the 45% reduction goal by 2027. However, under a moderate ambition scenario, the Dym‐EF projections are less optimistic than the static EF approach, with a delay of approximately five years (i.e., 2040 vs. 2035) in achieving the 25% reduction goal to limit global warming to below 2.0°C (Figure 1b). Additionally, both approaches conclude that the moderate ambition policies are incapable of reducing N_2_O emission by 45% before 2050.

The cumulative N_2_O emissions between 2011 and 2020 under current policies are found to be 9% higher than those projected under the moderate ambition policy and 26%–28% higher than those under high ambition policies (Figure 1c). Considering the long‐term effects, the timeline to meet the N_2_O reduction goal would be further delayed or missed. Both approaches consistently revealed that high‐ambition policies and strong climate action would lead to a 25% reduction in cumulative N_2_O emissions by 2022 and a 45% reduction by 2030–2032, approximately three to five years later than only considering the short‐term effects. In contrast, moderate ambition policies and medium climate action would only place us at the point of merely reaching the 25% reduction goal by 2050 or potentially missing the target. Given that no technology currently exists to remove N_2_O from the atmosphere, this finding underscores the urgency of early and deep N_2_O reduction. In the meanwhile, identifying the unmet goal will highlight the need for more aggressive measures targeting other sectors or other GHGs to counterbalance them.

### 3.2 Spatial Variability in Future N_2_O Emissions and Mitigation Potentials

**FIGURE 2:**
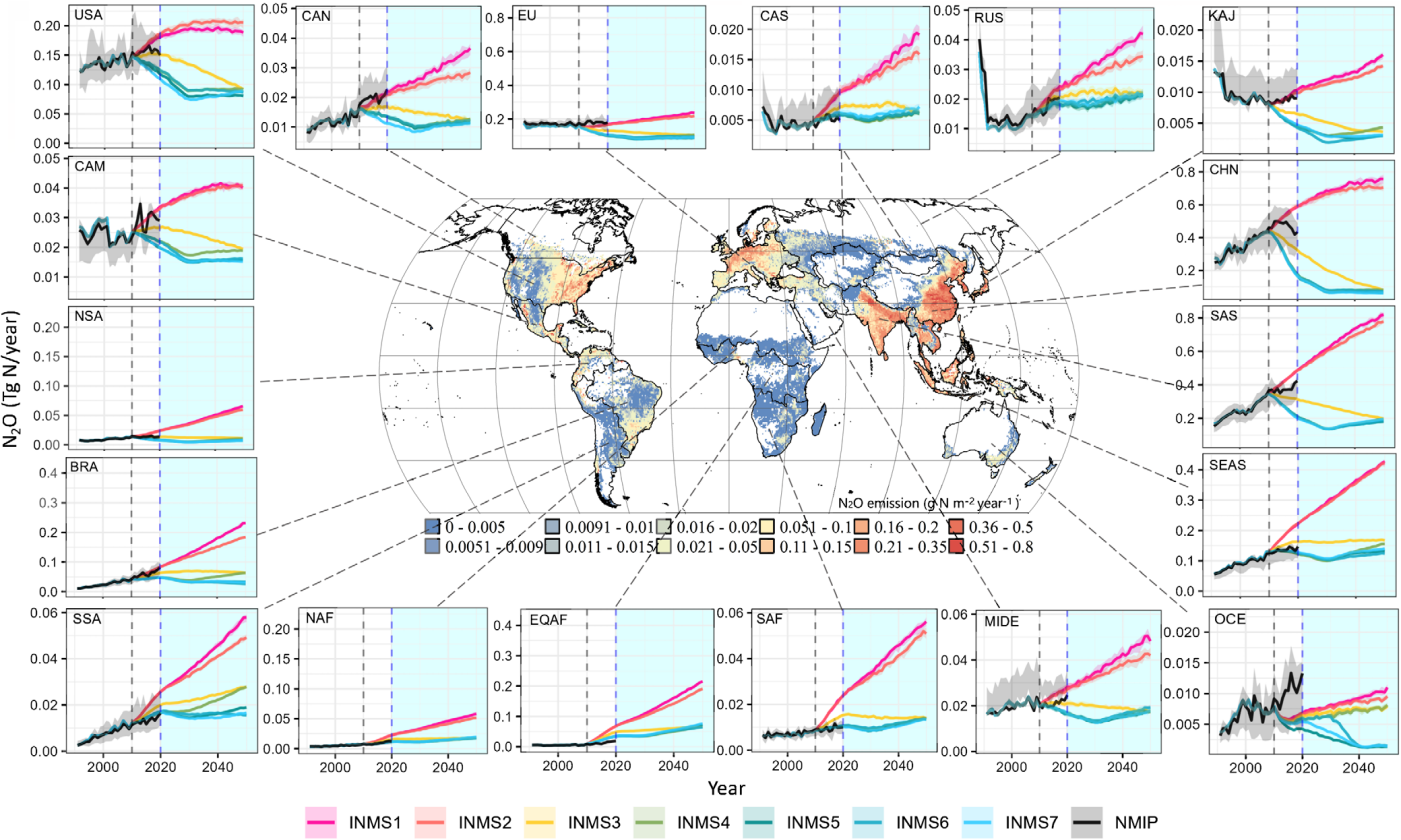
Projected N_2_O emission across different subregions during 1990–2050 based on the dynamic EFs. The black line represents the N_2_O emissions projected by the dynamic EF that reproduces the multi‐model ensemble. The shaded area for INMS1‐7 represents estimation range from the 25th to 75th percentiles driven by climate data from the 37 GCMs. The white and blue‐shaded time periods denote 1990–2020 and 2020–2050, respectively. The spatial map indicates the N_2_O emission estimated by NMIP2 ensembles in 2010 (unit: g N m^−2^ year^−1^). Key timeline markers include black and blue dashed vertical lines for the years 2010 and 2020, respectively. INMS1‐7 are depicted as seven INMS scenarios combining future climate change and N policies at different ambition levels. BRA, Brazil; CAM, Central America; CAN, Canada; CAS, Central Asia; CHN, China; EQAF, Equatorial Africa; EU, Europe; KAJ, Korea and Japan; MIDE, Mideast; NAF, Northern Africa; NSA, Northern South America; OCE, Oceania; RUS, Russia; SAF, Southern Africa; SAS, South Asia; SEAS, Southeast Asia; SSA, Southwest South America; USA, The United States of America.

**FIGURE 3:**
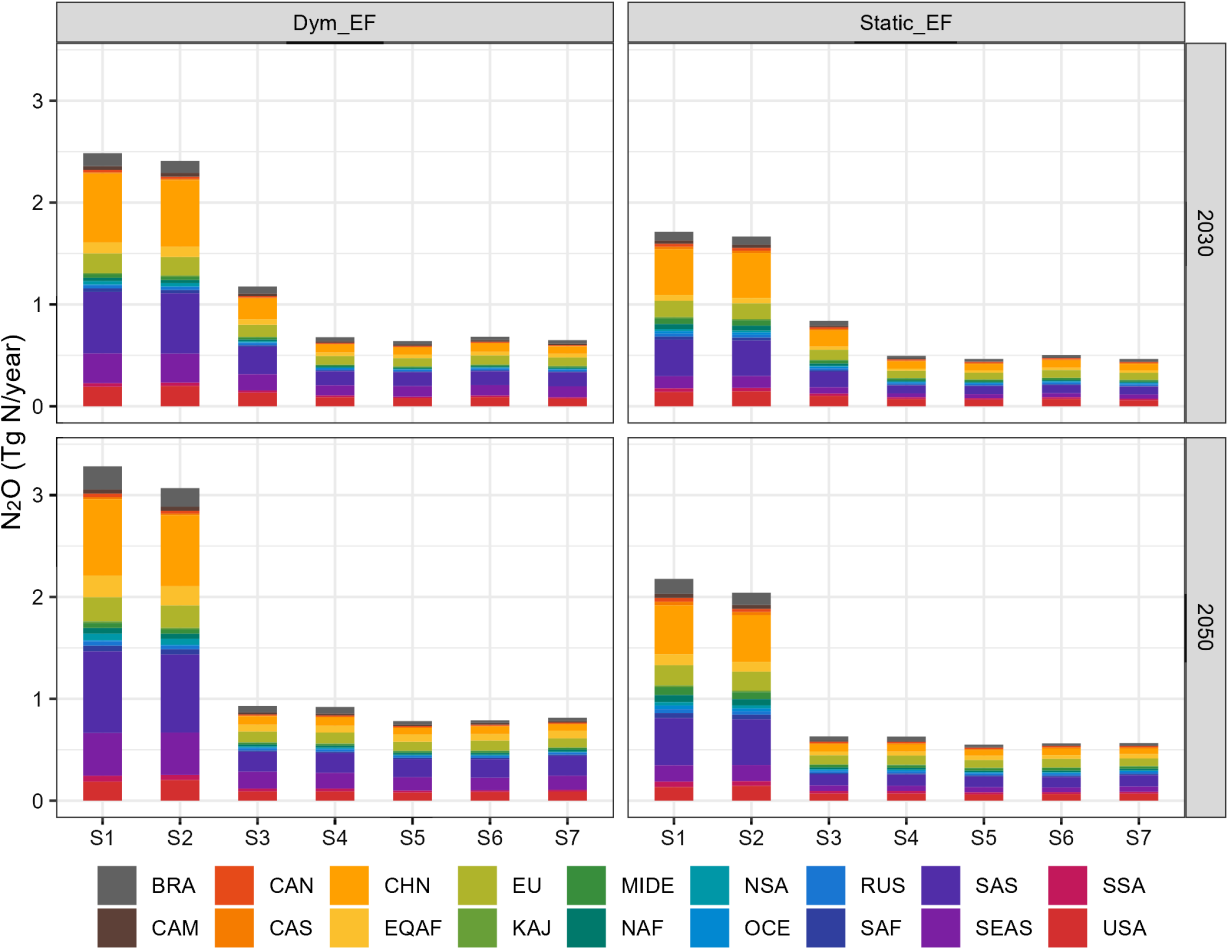
The total agricultural soil N_2_O emissions in 18 sub‐regions over the years 2030 and 2050, as projected by the Dym‐EF (left) and GAINS (right). The scenarios are labeled as S1 (INMS1), S2 (INMS2), S3 (INMS3), S4 (INMS4), S5 (INMS5), S6 (INMS6), and S7 (INMS7) representing a combination of N regulation policies at different ambition levels and climate action. BRA, Brazil; CAM, Central America; CAN, Canada; CAS, Central Asia; CHN, China; EQAF, Equatorial Africa; EU, Europe; KAJ, Korea and Japan; MIDE, Mideast; NAF, Northern Africa; NSA, Northern South America; OCE, Oceania; RUS, Russia; SAF, Southern Africa; SAS, South Asia; SEAS, Southeast Asia; SSA, Southwest South America; USA, The United States of America.

**FIGURE 4:**
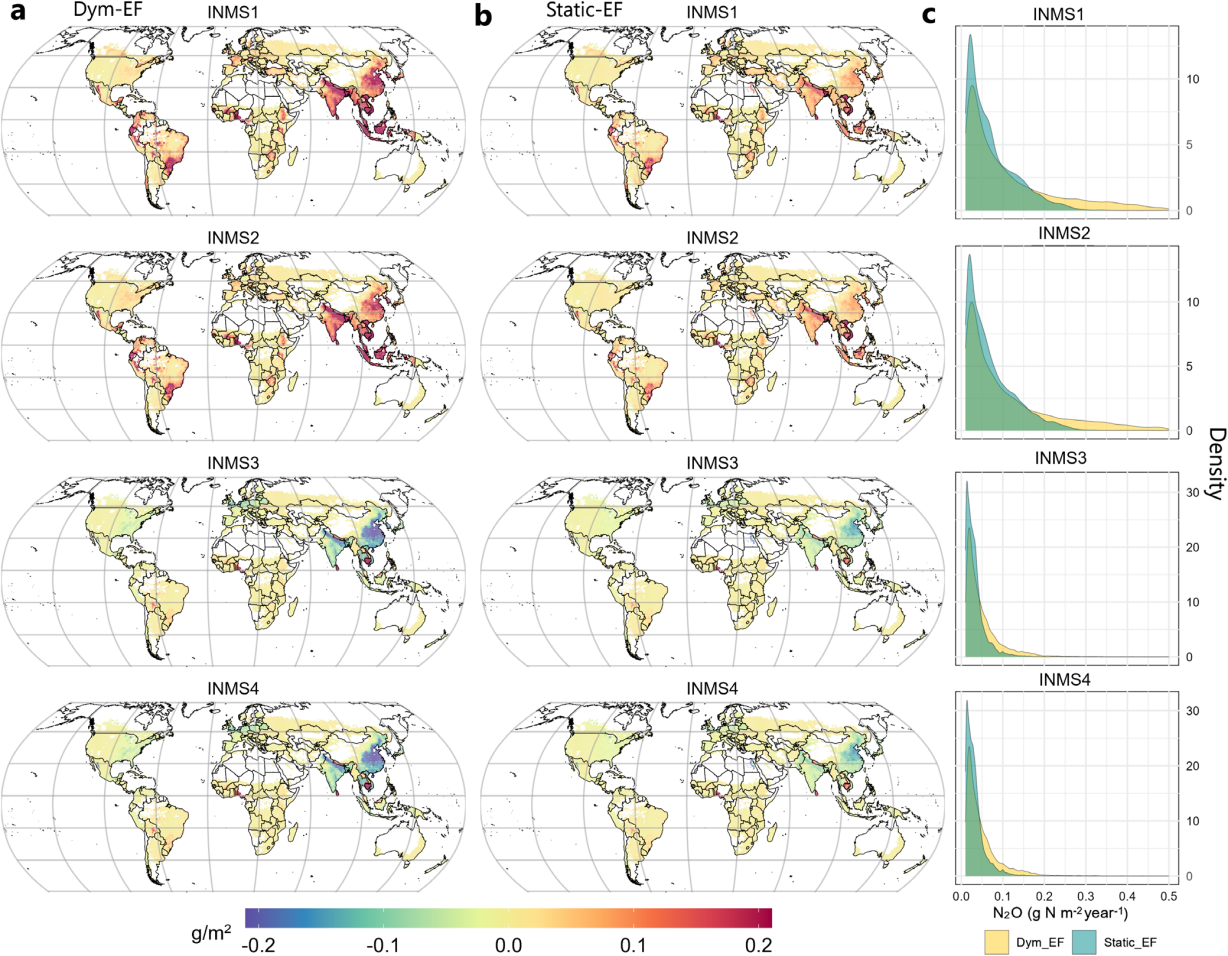
The projected N_2_O emission changes at global scales in 2050 relative to the 2010 baseline under scenarios INMS1 (BAU), INMS2 (Low N regulation), INMS3 (Moderate N regulation), and INMS4 (High N regulation). (a) N_2_O projections using the Dym‐EF. (b) N_2_O projections using the static‐EF. (c) Comparison of probability density distributions of global N_2_O Emissions in 2050 estimated by dynamic (Dym_EF, yellow) and static EF (Static_EF, blue) approaches for each scenario.

Our study shows that emission hotspots (defined as high N_2_O emission rate per unit land area) during the historical period will continue to be dominant source areas over the next three decades (Figures 2, 3, 4). Significant variations in future N_2_O emissions and their projected change trajectories exist across the 18 regions of global agricultural lands due to varying climate conditions, food demands, land productivity, and fertilizer inputs. Seven high‐emitting countries/regions, including South Asia, China, Southeast Asia, Brazil, the USA, the European Union, and Equatorial Africa, are expected to account for 83%–87% of global agricultural N_2_O emissions from fertilizer use by 2050 under all the examined scenarios (Figure 3).

Among these regions, recent‐decade N_2_O emissions in China, South Asia, and the USA under current policies (black lines with shaded area in Figure 2) fall between the projected levels under BAU/low‐ and moderate‐ambition N policy scenarios, indicating promising N_2_O mitigation potentials. Surprisingly, we find that the recent‐decade N_2_O emissions in Southeast Asia and Equatorial Africa are close to the levels projected under the high‐ambition policy scenario. It is likely because the NUE targets set for these two regions (Zhang et al. 2015) have been met or exceeded in the past decade, which led to a similar level or lower fertilizer input than was projected in the high ambition policy scenarios.

Despite NMIP2 estimations indicating a sharper decline in N_2_O emissions in China after 2013–2014 and in the USA after 2015–2016, which likely reflects the implementation of improved N management policies, these trends are less evident in other regions, and considerable uncertainty remains across the model ensembles. Additionally, while South Asia's agricultural N_2_O emissions track between low and moderate ambition levels, they show a dramatic increase during the 2010s, approaching the worst‐case scenario level by 2020. In Africa, despite currently low fertilizer use and limited cropland area, rapid population growth and rising food demand under BAU and low‐ambition policies are projected to drive higher N inputs in the future, causing a substantial increase in agricultural N_2_O emissions. Notably, regions like Canada, Russia, KAJ (Korea and Japan), Oceania, and Central America have a small share of global agricultural N_2_O sources under current policies, but their recent‐decade emissions are close to or even exceed those projected under low ambition scenarios, highlighting the need for deep and immediate reductions in these areas.

### 3.3 N_2_O Mitigation Potentials From Policy Shift

Globally, if N regulation policy and climate scenarios were shifted from BAU and low ambition to high ambition levels, we project that the N_2_O mitigation potential would reach 2.14–2.50 Tg N year^−1^ (min–max of the reductions among scenarios, a reduction of 70%–76% compared to the BAU and low‐ambition baseline) by 2050. This value is equivalent to 0.89–1.04 Pg CO_2_e year^−1^ (using 100‐year global warming potential of 256 to convert g N_2_O‐N to g CO_2_e, Myhre et al. 2014), which is averaged to 0.66–0.77 Mg CO_2_e ha^−1^ year^−1^ on global croplands. It is more than double the global soil carbon sequestration potential from planting of cover crops (estimated at 0.4 Pg CO_2_e year^−1^ by Bossio et al. (2020), assuming a soil carbon sequestration rate of cover cropping (Poeplau and Don 2015) at 1.17 Mg CO_2_e ha^−1^ year^−1^ on 400 Mha cropped area). Our findings suggest that reducing N_2_O emissions through fertilizer management on agricultural lands offers climate mitigation benefits comparable to other land‐based measures through soil carbon sequestration. In fact, it may provide even greater global benefits due to the current low NUE on extensive areas of croplands (Kanter, Winiwarter, et al. 2020). However, the static EF approach estimates a 35% lower N_2_O mitigation potential from the same policy shift (0.67 Pg CO_2_e year^−1^). This underestimation is primarily due to its inability to fully capture EF responses to climate warming and increased N inputs, leading to an underestimation of BAU/low‐ambition emissions (Figure 1). The N_2_O mitigation potential projected in this study (70%–76% by 2050) is substantially higher than previous estimates. For instance, earlier studies have reported mitigation potentials of 59% in cropland and grassland under the technical reduction and societal change scenario (UNEP/FAO 2024), 34% under implementation of 11 mitigation practices (Gu et al. 2023), and 47%–48% due to improved NUE and/or reduced fertilizer demand, relative to their 2050 BAU projections (Gao and Cabrera Serrenho 2023; Harmsen et al. 2019). Despite the range of scenarios examined in these studies, the core strategies for reducing N_2_O emission remain consistent: reducing fertilizer input and optimizing fertilizer management. Our higher estimates are primarily due to the inclusion of reductions in both fertilizer use and EF, accounting for the combined effects of N policy changes and climate scenario shift. In contrast, many previous studies either applied a static EF value or used N rate‐based EF, thus overlooking EF responses to climate changes. A recent study, using more environmental factors to inform a crop‐specific EF model (Cui et al. 2024, 2021), projected a 53% mitigation potential for improved fertilizer management in global croplands by 2020. While this EF model made progress in considering EF dynamics to factors beyond N input, it was based on experimental data with limited temporal coverage and did not take into account the growing fertilizer demand and long‐term climate change after 2020. These limitations may have contributed to a more conservative estimate of N_2_O abatement potential. While significant efforts are being made to explore and harness possible mitigation pathways across various GHG sources, this underestimation may diminish the perceived urgency and importance of developing policy incentives and technologies to lower agricultural soil N_2_O emissions.

Although different regions exhibit varying potential for reducing N_2_O emissions, the top contributing regions offer the greatest mitigation potential due to their high emission levels and opportunities to improve N management (Figure 3). For example, N_2_O emissions from the top seven source regions in 2050 could be cut from 2.67–2.83 Tg N year^−1^ to 0.65–0.77 Tg N year^−1^ if N fertilizer‐associated regulation policy and climate scenarios were shifted from BAU and low ambition to high ambition levels. This accounts for 76%–87% (min–max of the reductions among scenarios) of global N_2_O reduction potential if the policy shift can be implemented universally. It is important to note that some high‐ambition policies, such as the “best‐case” and “bioenergy” scenarios, rely on further reductions in N fertilizer use through strategies like dietary shifts (e.g., adopting low‐meat diets) and reducing food waste. While the feasibility of these strategies remains highly uncertain, our model projections show that in relatively low‐emission regions, such as Oceania, Southwest South America, and Central America, implementing the “best‐case” and “bioenergy” policies could lead to a substantial decrease in N_2_O emissions compared to the high N regulation scenario only (e.g., INMS4, Figure 2).

Among these regions, the policy shift will likely yield the largest N_2_O reduction benefit in China. Compared with the N_2_O emissions under the BAU scenario, implementing even moderate ambition policies could lead to a 70% reduction in China by 2030 and an 89% reduction by 2050. These figures are considerably higher than the global average N_2_O reduction potential under the same policy shift, which stands at 53% by 2030 and 72% by 2050. Moreover, even if moderate and high‐ambition policies are implemented in all countries, China's contribution to global agricultural N_2_O emission would substantially decline by 2050, from 23% under BAU and low ambition scenarios to 8%–9% (Figure S6). Other regions, such as Southeast Asia, the EU, and the USA, would become top source regions, only next to South Asia. It is likely because China, as one of the largest agricultural N_2_O emitter countries, is characterized by a higher fertilizer use rate and lower NUE than other countries/regions (Lu and Tian 2017; Tian et al. 2012; Zhang et al. 2015). Despite a dramatic decline in fertilizer input in China over the past decade, over‐fertilization still has been reported in croplands, especially for producing vegetables and fruits (Yu et al. 2022). Besides, much of China's cropping areas with intensive fertilizer input are concentrated in the Southeast of the country, exposed to subtropical and tropical climates, where EF changes between high‐ and low‐ambition policies, as well as strong‐ and weak climate action, are more pronounced than in other regions (Figure 4). Currently, these regions in China contribute significantly to global N_2_O hotspots. Worldwide, the top 10% of grid cells with the highest N_2_O emission rates—defined as hotspot areas—are projected to account for approximately 50% of total agricultural N_2_O emissions by 2050 under the BAU scenario. Similar above‐average N_2_O reduction potential under moderate N policies is projected in South Asia and Brazil, with reductions of 76% and 72% by 2050, respectively. The rest of the top source regions are projected to have relatively lower effectiveness of moderate ambition policies, with an N_2_O emission cut of 51%–68% compared with BAU. The below‐average policy effectiveness in these regions is likely due to the current high NUE levels relative to their target (e.g., high‐NUE OECD countries like the USA and the EU) or limited fertilizer reduction potential in non‐OECD countries with low N use, such as Southeast Asia and Equatorial Africa, under moderate N policies (Kanter, Winiwarter, et al. 2020).

The differences between static and dynamic EF approaches in projecting N_2_O emissions become more evident when we examine the magnitude and distribution of hotspot areas (Figure 4, Figures S7 and S8). Under BAU and low ambition scenarios, significant increases in N_2_O emissions are observed in southeastern China (CHN), SAS, SEAS, and parts of Equatorial Africa and Central America, primarily driven by substantial N inputs due to rapid population growth and a warming climate. However, the high N_2_O emission rates and rapid growth in these areas are largely underestimated by static EF‐derived estimations under the same climate and policy scenarios (Figure 4b). Based on the density plot, we find that the static EF approach yields more low‐N_2_O‐emission pixels and misses high N_2_O emission rates, particularly those exceeding 0.3 g N m^−2^ year^−1^, under the BAU and low ambition scenarios (Figure 4c). The underestimation of N_2_O emission hotspots and their rapid growth over time could potentially limit our efforts and investments in targeted N_2_O reductions, especially when compared to those measures directed at other GHG source sectors.

### 3.4 Low‐Hanging Fruits: Adopting N_2_O Reduction Technologies in Hotspot Areas

**FIGURE 5:**
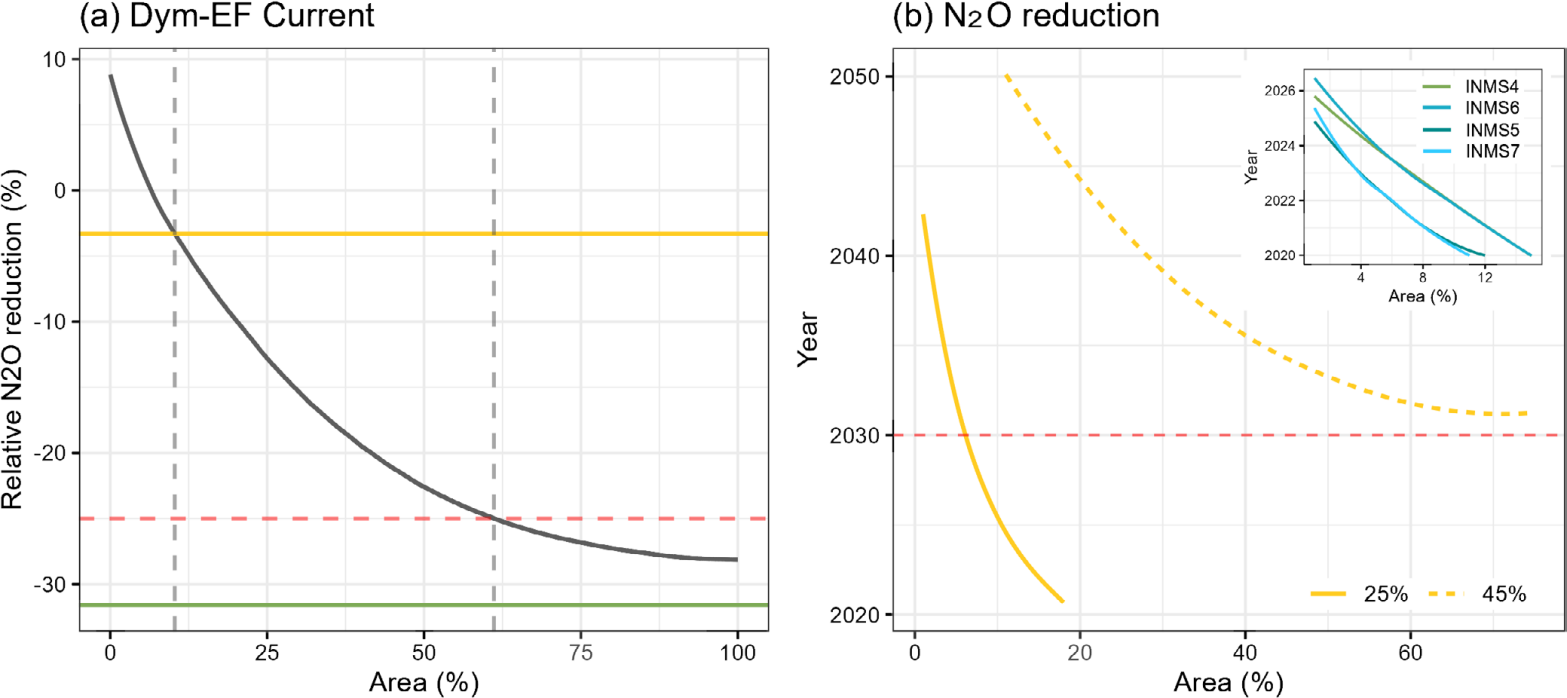
N_2_O mitigation potential projected by Dym‐EF by assuming the agricultural areas adopting nitrification inhibitors will be expanded along the hotspot gradient (ranked by N_2_O emission rate from per unit land area in each half‐degree grid cell). (a) Relative N_2_O Emission Reduction Under Current Policy (2020). The black line illustrates the percentage of N_2_O reduction potential if nitrification inhibitors are applied in 2020 with adoption area expanding in the half‐degree grid cells from the highest to lowest emission rates. The red dashed line represents a 25% reduction goal relative to the 2010 baseline. The yellow and green lines represent the projected N_2_O emission reduction under the INMS3 (medium N regulation) and INMS4 (high N regulation) scenarios in 2020, respectively, compared to the same baseline. (b) Years to achieve the 25% and 45% reduction goals when we assume the adoption areas of nitrification inhibitors are gradually expanded along the hotspot ranking under the moderate ambition policy scenario (yellow lines for INMS 3). The yellow dashed line represents the timeline to achieve the 25% reduction goal, while the yellow dotted line represents the timeline for the 45% reduction goal. The inset highlights the progress of high‐ambition policy scenarios (INMS 4–7) plus adoption of N_2_O reduction technology in achieving the 45% emission reduction target.

We evaluated the effectiveness of nitrification inhibitor (NI) adoption by prioritizing hotspot areas, ranked by the projected N_2_O emission rates per square meter land in each half‐degree grid cell (using historical N_2_O estimate under current policies in 2020 as an example, Figure S9), and then gradually expanding the extent of NI adoption. We chose NI as a tested practice, instead of other N reduction strategies, to avoid double counting their impacts under the moderate‐to‐high ambition N regulation policies. Here, the NI effectiveness has been quantified by examining N_2_O reduction only without changing N fertilizer use rate or cost (details in Methods). Initially, the N_2_O reduction percentage rises rapidly along the hotspot gradient but stabilizes as the area of NI adoption grows. This highlights the importance of targeting hotspot areas for N_2_O reduction technologies. Under current policies, applying NI to the top 10% hotspot area could reduce the 2020 N_2_O emissions to levels projected under moderate ambition policies. To achieve the 25% reduction goal (dashed line in Figure 5a), NI would need to be applied to 63% of agricultural lands along the hotspot ranking. Under moderate nitrogen regulation and climate scenarios, we project that N_2_O reductions will fall short of the 25% goal before 2030. However, incorporating NI could accelerate progress toward this target. For instance, adding NI to 8% or more of top hotspot areas could enable us to meet the 25% reduction target by 2030 or earlier, even under moderate ambition policies (dashed yellow line in Figure 5b).

Regarding the 45% reduction goal, our findings are less encouraging. Even with the addition of NI in the top hotspot areas, achieving a 45% reduction before 2030 is unfeasible for moderate ambition policies and climate scenarios (the dotted yellow line in Figure 5b). Note that the mitigation potential reported here should be interpreted as the effect of scenario transitions that combine climate action and nitrogen policies rather than the effect of a single mitigation practice. In addition, the NI scenario in this study represents an idealized implementation in which the assumed mitigation efficiency is uniformly applied to all targeted grid cells, without accounting for adoption barriers, management constraints, or site‐specific variability in effectiveness. Consequently, the estimated additional mitigation potential does not represent a realistic deployment outcome. Instead, it illustrates the critical role of hotspot areas in achieving N_2_O reduction goals and highlights the nonlinear gains that may be realized by sequentially adopting additional mitigation practices based on hotspot prioritization. This result underscores the urgency of shifting current N regulation policies to high ambition levels and reducing N_2_O emissions in hotspot areas to align with the GHG reduction target to control global warming below 2°C.

### 3.5 Limitations and Uncertainties in This Work

Despite progress made in capturing the EF dynamics in response to multiple environmental drivers, this study still has several limitations. First, we exclusively investigated global N_2_O emissions from scenarios that combine future climate change and policy interventions that affect N fertilizer inputs. N_2_O emissions from animal manure were not examined in this work. However, it could be another important component affecting the agricultural emission of N_2_O (Charles et al. 2017; Walling and Vaneeckhaute 2020). With improved NUE and constant or increased availability of manure due to improved handling, the need for mineral fertilizer in crop production could decrease significantly. The agricultural soil N_2_O mitigation potential reported here (70%–76% due to climate action and policy shift regarding N fertilizer inputs) is likely to be smaller if manure N management is also considered. Future work should explore the potential agricultural N_2_O changes considering the assumptions of future manure use, under various dietary structures and land use distribution among food‐feed‐energy production, while also accounting for the dynamics of EFs. Second, to avoid the risks of double‐counting mitigation potential and overusing the Dym‐EF approach, we did not specifically examine the impacts of additional conservation practices, such as reduced tillage, cover crops, and biochar applications, on N_2_O emission dynamics (Gu et al. 2023; Lehmann and Joseph 2024; Six et al. 2004). Many of these practices contribute to improving NUE and reducing fertilizer input, effects that are already captured in the optimized N policy scenarios. Moreover, the EF training data from the NMIP2 simulations did not include these practices as input drivers. To date, these practices have been adopted in very limited areas of global croplands. Unless the effects of emerging technologies on further reducing N_2_O emissions at a fixed level of fertilizer use are considered, our projections may represent an upper bound of mitigation potential. This is based on the fact that increasing fertilizer demand, expansive cropland areas, and a warming climate are the primary drivers of future N_2_O emissions. Compared with commonly used static‐EF methods, Dym‐EF effectively captures spatial and temporal EF variability driven by climate, soil, and management, but it still depends on the available data from underlying process‐based simulations. In its current implementation, Dym‐EF does not explicitly represent crop‐specific or management‐specific responses, as the NMIP2 participant models do not yet provide direct soil N_2_O estimates differentiated by crop types or conservation practices. We anticipate future work incorporating more detailed information on crop types and management practices, particularly from data‐rich regions, could further enhance the development and applicability of Dym‐EF.

Last but not least, our uncertainty quantification only reflects the projection uncertainties derived from climate input data estimated by 37 GCM models. Considering the uncertainties associated with other input data, such as future N input patterns and trends across countries, is likely to yield a wider estimate range. The future fertilizer input, used to drive the N_2_O projection in this study, was developed based on country‐level food demand forecasts and different trajectories of NUE improvement. These future fertilizer trajectories should be interpreted as scenario‐based and illustrative rather than prescriptive forecasts. To align with the NUE targets proposed by Zhang et al. (2015), the INMS framework, which defines country‐ or region‐level fertilizer trajectories, including when and how rapidly these targets are approached, under low‐, medium‐, and high‐ambition policy scenarios (Kanter, Winiwarter, et al. 2020). Since the INMS framework does not provide a formal uncertainty analysis, uncertainty associated with these scenario assumptions was not quantified separately in this study. In addition, Zhang et al. (2015) did not provide separate NUE targets for specific agricultural land categories, such as croplands or pasture, pasture fertilizer inputs were not assigned an independent pathway but instead followed the same country‐ or region‐level fertilizer scaling. These NUE targets are not prescriptive for specific countries but are intended to illustrate the levels of efficiency required to meet projected food demand while keeping N surplus around 50 Tg N year^−1^, in line with planetary boundary thresholds. For example, the target NUE is set at 0.75 for the EU and USA, 0.60 for China and much of Asia, and 0.70 for other regions. Given the substantial variability in actual NUE across crops, countries, and regions, achieving these targets will likely require a combination of technological and management interventions. In addition, these input patterns are influenced by factors such as socioeconomic development, policy changes, international cooperation and trade, which remain challenging to project the future robustly and should be addressed with more advanced scenario designs (Mosier et al. 1998; Smith et al. 2013; Vishwakarma et al. 2022). The mitigation potentials reported here should therefore be interpreted as scenario‐based estimates rather than probability‐based predictions. Future research is needed to assess the feasibility of meeting these NUE targets at the national level and to evaluate how different NUE improvement trajectories—particularly those involving or excluding key advancements such as breeding techniques that enhance yield response to N additions (Vishwakarma et al. 2022)—could influence the overall N_2_O mitigation potential under policy shifts.

### 3.6 Implications for Future Mitigation Policies

The heavy burden of meeting growing food, feed, and biofuel demands, coupled with the present dietary structures and low crop NUE, poses significant challenges in reducing agricultural N_2_O emissions globally (Godfray et al. 2010). Our study suggests that high‐ambition N regulation policies aimed at national‐level NUE improvement by 2030 offer the potential to significantly lower agricultural N_2_O emissions, thereby easing the GHG mitigation pressures on other sectors in efforts to limit climate warming below 2°C. In addition, we should also recognize the spatial heterogeneity and cross‐crop difference in N_2_O intensity of crop production across the world (Carlson et al. 2016). Improved international cooperation to enhance whole system NUE and reduce food loss and waste, as well as identifying alternative food and renewable energy sources (Gallon et al. 2022; Kanter, Chodos, et al. 2020; Lewis and Nocera 2018), could cut N_2_O emissions while meeting the growing demands of worlds' population. A wide variety of recent studies have explored the N_2_O reduction potential of new technologies (Jules et al. 2018), nature‐based or bioengineering‐derived mitigation practices, policy options, and landscape redesign, with a focus on reducing fertilizer use (Xiao et al. 2024; Yang et al. 2024), enhancing plant‐microbial interactions and plant NUE (Zhang et al. 2021), or cultivating N_2_O‐consuming bacteria (Hiis et al. 2024). These haven't been examined in this study but could possibly alleviate the pressure of adopting more aggressive N regulation policies in the future. Furthermore, integrating precision agriculture technologies, such as satellite‐ and airborne‐based monitoring and machine learning‐based decision‐making systems, into farming practices holds a promise to optimize N application, enhancing NUE and reducing N_2_O emissions (Berger et al. 2020; Bossio et al. 2020). There is also a need for comprehensive assessment and identification of effective policies that incentivize sustainable agricultural practices and promote research and development in NUE‐enhancing technologies. Farm‐level conservation practices like better N management, reduced tillage, cover crops, and biochar application are likely to significantly mitigate N_2_O emissions by improving soil health and reducing direct N_2_O emissions and nitrogen runoff (Dueri et al. 2023; Jonassen et al. 2022; Lu et al. 2022; Pan et al. 2022). National‐level and international socio‐economic changes ought to be considered to ensure that smallholder farmers are supported in the transition to sustainable practices. International cooperation is essential to share knowledge, technologies, and resources, aiming for a global reduction in N_2_O emissions and achieving the United Nations SDGs.

## 4 Conclusion

This study contributes to the ongoing effort to improve future projections of agricultural N_2_O emissions by employing a novel dynamic emission factor (Dym‐EF) framework, which integrates machine learning techniques with process‐based models. Unlike conventional static emission factor approaches, our dynamic methodology captures non‐linear and spatially heterogeneous responses of N_2_O emissions to climate change, soil properties, and varying fertilizer application rates. This work aligns with recent findings from the Global Nitrous Oxide Assessment, which emphasizes the urgent need for ambitious and integrated N management strategies, particularly within the agricultural sector, to address rapidly increasing N_2_O emissions globally. More importantly, this study highlights the need to capture EF dynamics into robust emission projections. Compared to the widely used static EF approach, we project higher soil N_2_O emissions from agricultural lands and larger mitigation needs under future climate conditions. Through scenario‐based analyses, we quantify the substantial mitigation potential achievable by adopting targeted, high‐ambition N management policies. This approach enhances our understanding of agricultural N_2_O emission trajectories and informs policy strategies aligned with international climate targets.

## Author Contributions

**Wilfried Winiwarter:** conceptualization, funding acquisition, writing – review and editing, methodology, resources, project administration, investigation. **Josep G. Canadell:** resources, writing – review and editing, funding acquisition, project administration. **Chaoqun Lu:** conceptualization, investigation, funding acquisition, writing – original draft, methodology, supervision, resources, project administration, formal analysis, writing – review and editing, software. **Linchao Li:** investigation, methodology, validation, visualization, writing – review and editing, writing – original draft, formal analysis, software, data curation. **Hanqin Tian:** project administration, resources, writing – review and editing, funding acquisition. **Weihang Liu:** methodology, validation, writing – review and editing, formal analysis, visualization, data curation.

## Funding

This work is supported by the OECD Co‐operative Research Program fellowship, USDA AFRI (2023‐67019‐39252), NSF Grant (1903722 and 1945036), and by European Union's Horizon Europe Research and Innovation programme (#101081395, EYE‐CLIMA).

## Conflicts of Interest

The authors declare no conflicts of interest.

## Supporting information


**Appendix S1:** gcb70919‐sup‐0001‐Supinfo.docx.

## Data Availability

The projected annual global N_2_O emissions from agricultural soils supporting this study's findings are available in Figshare at https://doi.org/10.6084/m9.figshare.32194662. The projected N_2_O EF under seven scenarios is available in Figshare at https://doi.org/10.6084/m9.figshare.26384401.
