# Supplementary material for: Underestimation of Future Agricultural Soil N2O Emissions and Abatement Needs
Source: Glob Chang Biol. 2026 May 15;32:e70919. doi: 10.1111/gcb.70919 (PMC13178208; doi:10.1111/gcb.70919)
Supplement: Supplementary file 1 — Appendix S1: gcb70919‐sup‐0001‐Supinfo.docx. [file GCB-32-e70919-s001.docx]

Supplementary materials for

**Underestimation of future agricultural soil N₂O emissions and abatement needs**

Chaoqun Lu^1,2*^, Linchao Li^2^, Wilfried Winiwarter^3,4^, Josep G Canadell^5^, Weihang Liu^2^, Hanqin Tian^6,7^

^1^ Department of Soil and Environmental Sciences, University of Wisconsin-Madison, Madison, WI 53706, USA

^2^Department of Ecology, Evolution, and Organismal Biology, Iowa State University, Ames, IA 50011, USA

^3^ International Institute for Applied Systems Analysis, A-2361 Laxenburg, Austria

^4^ Institute of Environmental Engineering, University of Zielona Góra, 65-417 Zielona Góra, Poland

^5^ CSIRO Environment, Canberra, ACT, Australia

^6^ Center for Earth System Science and Global Sustainability, Schiller Institute for Integrated Science and Society, Boston College, Chestnut Hill, MA 02467, USA

^7^ Department of Earth and Environmental Sciences, Boston College, Chestnut Hill, MA 02467, USA

*Correspondence to clu274@wisc.edu

**Evaluating the performance of Dym-EF**

The NMIP2 participant models underwent calibration and validation prior to global simulations. Their N₂O estimates were evaluated against a range of benchmark datasets, including site-level and tower-based emission measurements, statistical extrapolations, and atmospheric inversion results ^1^. The estimates were also integrated with emissions from non-terrestrial sources, such as industry, combustion, wastewater, inland waters, and ocean, to support assessments of the global N₂O budget and to help explain observed trends in atmospheric N₂O concentrations ^2^. Built upon the NMIP2 model ensemble, we assessed the consistency between Dym-EF and NMIP2 ensemble median in estimating EF dynamics across global croplands and over time ^3^. In this study, we further evaluated the performance of Dym-EF by comparing the EF estimates with observations from multiple sites across the world. In the global N_2_O database, most of them measured N_2_O emissions during partial or whole growing season, while process-based models like NMIP2 estimate N_2_O emissions for an entire year. To eliminate this difference, we calculated the ratio of N_2_O EF during growing season and annual period based on a global meta-analysis ^4^, which contains the records of measurement duration. We separated the seasonal and annual records using a threshold of 300-day measurement within a year. Growing season measurement records are more than double annual records in this database. The results indicate the growing-season N_2_O EF is about 75% (72–86%) of annual N_2_O EF (Figure S1).


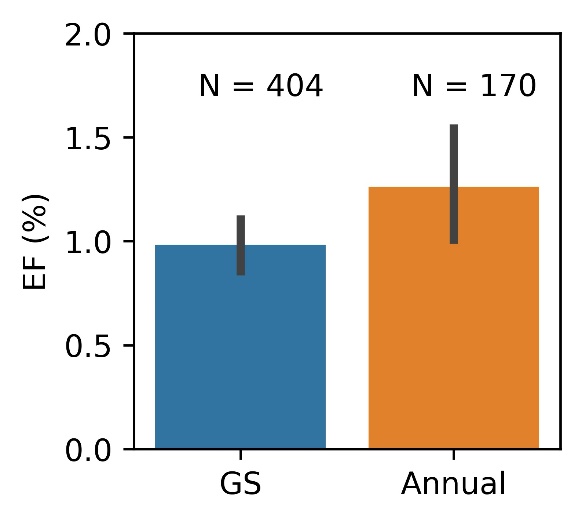


Figure S1 The observational EF based on N_2_O measurement within a growing season (or less than a full year) and annual period, which is separated by a threshold of 300-day measurement per year. GS: growing seasonal scale, Annual: annual scale.

We then validated the simulated N_2_O EF we used in this study with the Global N_2_O Dashboard & Database (<https://samples.ccafs.cgiar.org/n2o-dashboard/>). It is an up-to-date database of field measurements of N_2_O emissions based upon studies collected by Stehfest & Bouwman, (2006) ^5^ and extensive studies published more recently (Figure S2). To be included in the final database, a study must meet the following requirements: (1) N₂O emissions must be reported from at least one unfertilized control experiment and one fertilized treatment, and (2) it must provide both the amount of N fertilizer applied and the cumulative N₂O emissions measured over the trial period. These criteria facilitate calculating the observed EF at multiple sites.


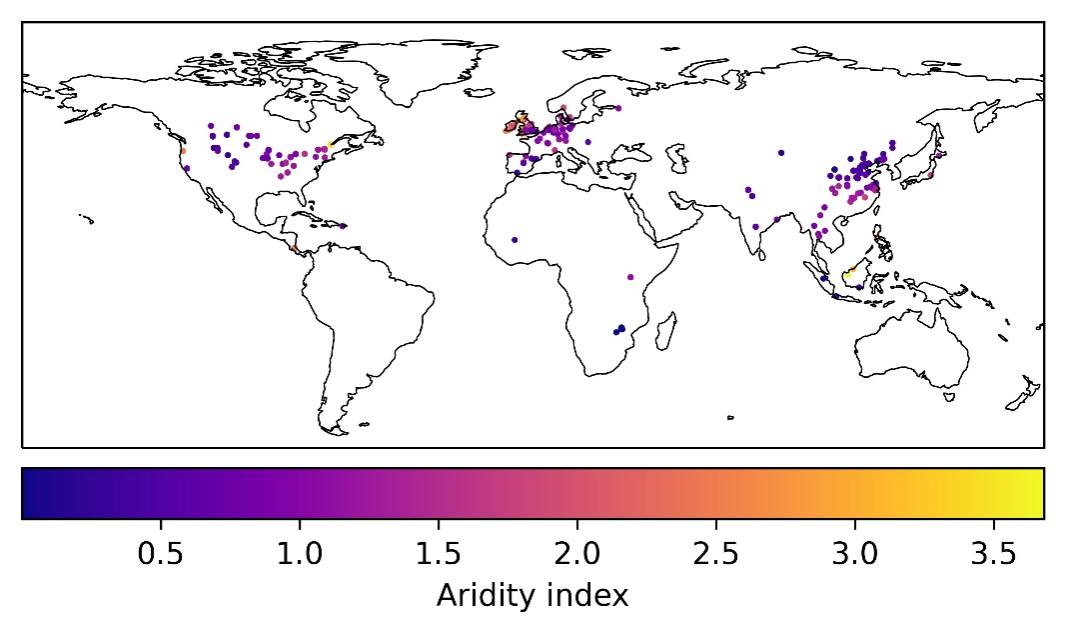


Figure S2 The locations of N_2_O measurement sites with EF reported (from Global N_2_O Dashboard & Database, <https://samples.ccafs.cgiar.org/n2o-dashboard/>).

Since the temporal scales of simulated and observed N_2_O EF are different, we use the ratio (1/0.75) derived from the meta-analysis to correct the observational N_2_O EF in growing season to annual EF. When comparing the simulated EF from Dym-EF and observed N_2_O EF, we grouped the measurement sites based on multi-year average aridity index. The annual aridity index is defined as the P/PET, and the raw data are obtained from 0.5°×0.5° CRU ^6^. The comparison indicates that the simulated EF matches with observed EF along the AI gradient, with a RMSE of 0.3% (Figure S3). Similarly, the simulated EF is found to match well with observed EF along SOC content graident (Figure S3). The wide EF range from measurement data reflect the variability of environmental factors, such as soil properties, and various management practices across sites. In contrast, half-degree simulation data have aggregated the spatial details and demonstrate smaller cross-site variations.


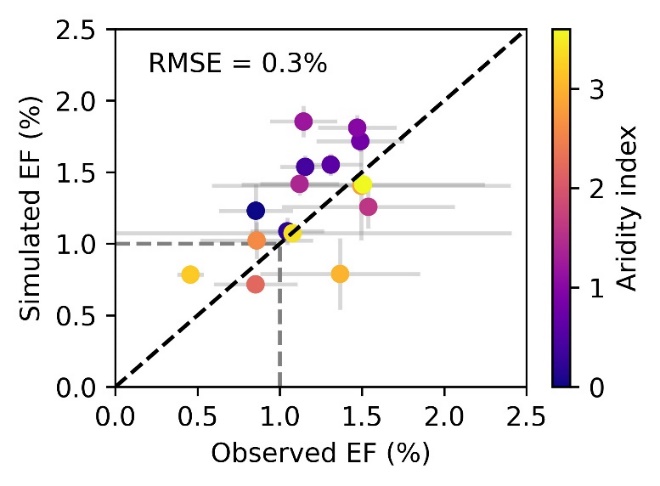

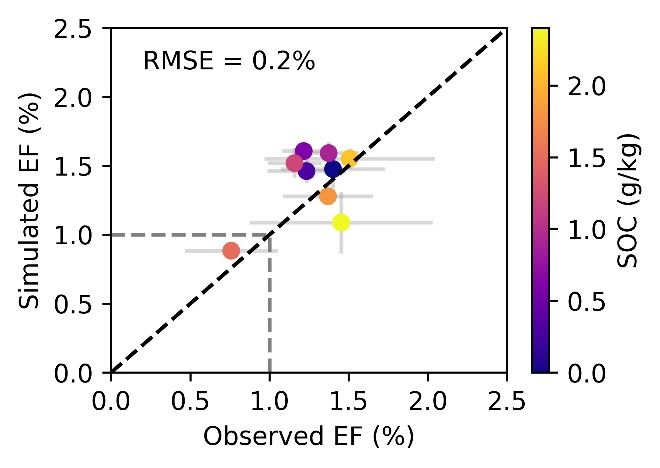


Figure S3 The comparisons between observed and simulated EF, with observation sites grouped by aridity index (AI, left) and by soil organic carbon content (SOC, right). Observation sites are grouped by intervals of 0.2 for AI and 0.3 for SOC content. The dots represent the average EF and the error bars are the 95% confidence interval within each AI and SOC bin. The gray dash lines indicate the default Tier-1 EF defined by IPCC guideline.

Generally, the Dym-EF model demonstrates strong performance in reproducing N_2_O emission dynamics simulated by the NMIP2 multi-model ensemble. The temporal dynamics of N_2_O emissions estimated by Dym-EF closely align with the NMIP2 ensemble median over the period 1990–2020, indicating that the machine learning model effectively captures the nonlinear relationships between EFs and key environmental drivers. After 2010, the increasing rate of fertilizer-induced N_2_O emissions appear to slow down in both estimations, which might be attributed to improvements in nitrogen management practices, such as enhanced fertilizer use efficiency or reduced application rates in certain regions. However, it is important to note that the N_2_O emission estimates in this study solely reflect the N_2_O source resulting from climate change and synthetic nitrogen fertilizer inputs. While the overall trends from Dym-EF and NMIP2 are broadly consistent, some differences remain. For example, the NMIP2 estimates are derived directly from eight process-based terrestrial biosphere models, each with distinct structures, parameterizations, and process representations, leading to a broader estimate range (Fig. S4). In contrast, although the training data for the Dym-EF model originate from multi-model NMIP2 simulations, the model only learns the median values of NMIP2 ensemble and therefore doesn’t reflect the full range of structural differences across NMIP participant models (Figure S4). It is noteworthy that the median values of NMIP2 ensemble demonstrate a nearly-flat pattern of N_2_O emissions during the last decade, while the model ensemble mean shows a slight increase in this same period ^1^. We used the ensemble median EF as training data for developing the Dym-EF, thereby reducing the influence of overweighted results or outlier from individual models.


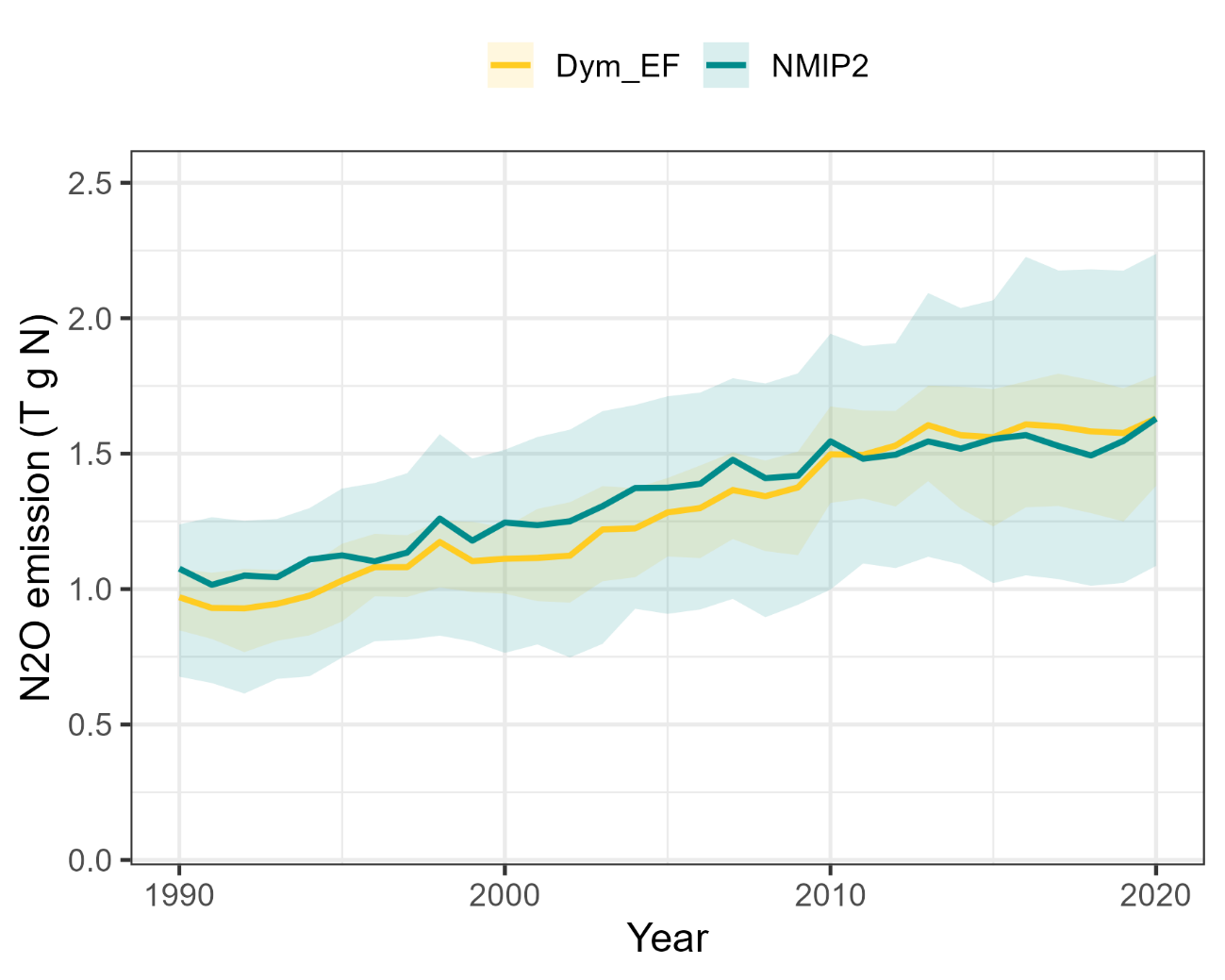


Figure S4 The comparison of agricultural fertilizer use-associated N_2_O emission between Dym-EF and NMIP2 estimations from 1990 to 2020. Shaded area represents 95% confidence interval of each approach.

**Environmental and management factors regulating N_2_O EF (Emission Factor) dynamics**

The analyses summarized here build on our previous study of the Dym-EF framework^3^, but are briefly reintroduced here to provide additional context for the environmental controls represented in this study. To better interpret the environmental controls represented by the Dym-EF framework, we analyzed predictor importance and marginal response patterns using Shapley additive explanations (SHAP) and partial dependence plots (PDPs). The Dym-EF model was trained to emulate the multi-model median EF from the NMIP2 ensemble using climate, soil, and management variables at 0.5° resolution. This analysis helps explain why projected EFs vary across regions and scenarios.

The variable-importance analysis shows that EF dynamics are mainly controlled by the interaction of nitrogen management and seasonal climate. Summer temperature (T_JJA), nitrogen fertilizer input (NFer), summer precipitation (Pr_JJA), and autumn precipitation (Pr_SON) are the most important predictors. Soil properties generally play smaller roles, although soil pH is more influential than other static soil variables.

The SHAP analysis further indicates that the dominant controls on EF vary across space and time. In historically high-input regions, such as Southeast Asia, Central Europe, the US Corn Belt, and Southwest China, the dominant driver shifts from fertilizer input or spring/autumn temperature in earlier periods toward summer temperature and precipitation in later periods. This suggests that EF becomes increasingly sensitive to climate variability as warming intensifies and fertilizer use increases. In contrast, in historically low-input regions, especially in parts of Africa and South America, fertilizer input becomes more important over time, reflecting the growing influence of anthropogenic N additions.

The PDP analysis reveals strong nonlinear responses. EF generally increases with nitrogen fertilizer input, but the increase weakens at higher application rates, indicating a saturating response. Temperature also has a positive effect on EF, with stronger responses in summer and autumn once temperatures exceed roughly 2–6°C. Precipitation shows a threshold-type relationship: EF increases with seasonal precipitation up to a certain level and then stabilizes, consistent with the role of soil moisture in promoting nitrification and denitrification.

Among soil properties, soil pH shows the clearest marginal relationship with EF. EF generally decreases as pH increases above about 5.0–5.3, suggesting that moderately acidic soils are associated with higher EF values. Other soil variables, including texture, bulk density, and soil organic carbon, show weaker and more complex effects.

Overall, the SHAP and PDP analyses show that the Dym-EF model captures process-relevant controls on EF. While EF is jointly regulated by N input and climate, the effects of temperature and precipitation are nonlinear, and dominant controls can shift across regions and over time. These results support the use of a dynamic EF approach for future projections, as static EF methods cannot represent these nonlinear and spatially heterogeneous responses.

**Comparison between static and dynamic EF-based N_2_O projections**

Static EF approach yields a larger N₂O reduction potential under moderate to high ambition scenarios and a smaller N₂O increase under low ambition scenarios than dynamic EF modeling (Fig. 1). Using the Dym-EF model, we project that policy shift from BAU/low ambition to high ambition levels would cut N_2_O emissions from the top seven source regions by approximately 2 Tg N yr^-1^ by 2050. This accounts for 76-87% (min-max of the reductions among scenarios) of global N₂O reduction potential if the policy shift can be implemented universally. However, the importance of hotspot regions is underestimated by the static EF approach, which concludes that these seven regions will account for 75-79% of the global total emissions by 2050 (vs 83-87% from Dym-EF, Fig. 3), and likely contribute 70-78% of global N₂O reduction potential from the same policy shift.

The static EF approach underestimates the share of South Asia, Southeast Asia, Equatorial Africa, and Central America to the global total N₂O emission across all the scenarios (Figure S6). This underestimation occurs because the EF changes in response to climate and N inputs are more pronounced in tropical areas, which is not reflected in the static approach ^3,7^. In addition, we find the static EF approach underestimates the N₂O reduction potential between BAU/low and moderate/high ambition policies, especially in the top source regions (Fig 2 and Figure S7).

**N_2_O mitigation potential of moderate ambition N policies**

This study confirms the significant benefits of adopting high-ambition N regulation policies early and robust climate actions. However, moderate-ambition N regulation policy and climate actions are also effective in reducing N_2_O emissions at a lower cost. The projections from Dym-EF show that, even under moderate-level N regulations and climate action scenario (SSP2-4.5), agricultural N₂O emissions are still projected to decline, reaching 1.18-1.21 Tg N yr^-1^ by 2030 and 0.93-0.96 Tg N yr^-1^ by 2050, 15-19% and 33-35% lower than the 2010 level, respectively. If N policies and climate actions were shifted from BAU/low ambition to moderate ambition level, N₂O emissions from these seven regions could be reduced to 1.01 Tg N yr^-1^ by 2030 (i.e., approximately half of the N₂O level under the BAU and low ambition scenarios), and 0.77 Tg N yr^-1^ by 2050 (nearly a three-quarter reduction). Our projections show that shifting from low-ambition to moderate-ambition policies, the least N₂O reduction would be found in Oceania (13% reduction by 2030 and 29% by 2050) and Russia (29% by 2030, and 48% by 2050). This is primarily due to the lower EF and relatively lower fertilizer use in these regions with different baseline emissions profiles ^8–10^. However, it does not mean there is limited or no need for these regions to implement moderate or high-ambition policies.

Table S1 Descriptive summary for nitrogen policy scenarios with different ambition levels that are associated with synthetic N fertilizer input (Modified from Kanter et al.^11^)

| Country group | Nitrogen Policy Ambition Levels | | |
| --- | --- | --- | --- |
|  | **High** | **Medium** | **Low** |
| **OECD** | Target NUE by 2030 | Target NUE by 2050 | Current NUE remains constant |
| **Non-OECD /Moderate-High N*** | Target NUE in 10 years after catch-up^$^ with OECD countries | Target NUE in 30 years after catch-up with OECD countries | NUE trends are expected to improve or remain constant. If the negative trends from the past 10 years persist, they will continue until 2030; otherwise, NUE is projected to stabilize. |
| **Non-OECD/Low N^#^** | Target NUE in 30 years after catch-up by avoiding historically polluting N trajectory of other countries. It is assumed they will “tunnel through” from low input/high NUE to moderate input/high NUE over a 30-year period | NUE follows historical N trajectory towards high N/low NUE over 30 years, before improving | Current decreasing NUE trends continue akin to countries with similar socioeconomic status and are expected to stabilize by 2030 at the latest. |

*Moderate to high N use is defined as an N surplus greater than 50 kg N ha^−1^, e.g. China, based on data from Zhang et al. (2015)^12^. ^#^non-OECD countries with low N use are defined as N surplus less than 50 kg N ha^−1^, e.g. Malawi. ^$^ “Catch-up” refers to non-OECD countries reaching high-income levels, a criterion that changes over time. For example, the threshold in 2010 was 12,275 USD/capita/yr according to World Bank data.

**Table S2** The seven future climate, land use, diet, and N management scenarios.

| **Name** | **Scenario** | **Climate** | **Land use regulation** |  | **Productivity** | **Diet** | **Ambition level** |
| --- | --- | --- | --- | --- | --- | --- | --- |
| INMS1 | Business-as-usual | SSP585 | Medium |  | High | Meat & dairy-rich | Low |
| INMS2 | Low nitrogen regulation | SSP245 | Medium |  | Medium | Medium meat & dairy | Low |
| INMS3 | Medium nitrogen regulation | SSP245 | Medium |  | Medium | Medium meat & dairy | Moderate |
| INMS4 | High nitrogen regulation | SSP245 | Medium |  | Medium | Medium meat & dairy | High |
| INMS5 | Best-case | SSP245 | Strong |  | High | Low meat & dairy | High |
| INMS6 | Best-case “Plus” | SSP245 | Strong |  | High | Ambitious diet shift and food loss/waste reductions | High |
| INMS7 | Bioenergy | SSP126 | Strong |  | High | Low meat & dairy diet | High |

*^*^Modified from Kanter et al. (2020b).*


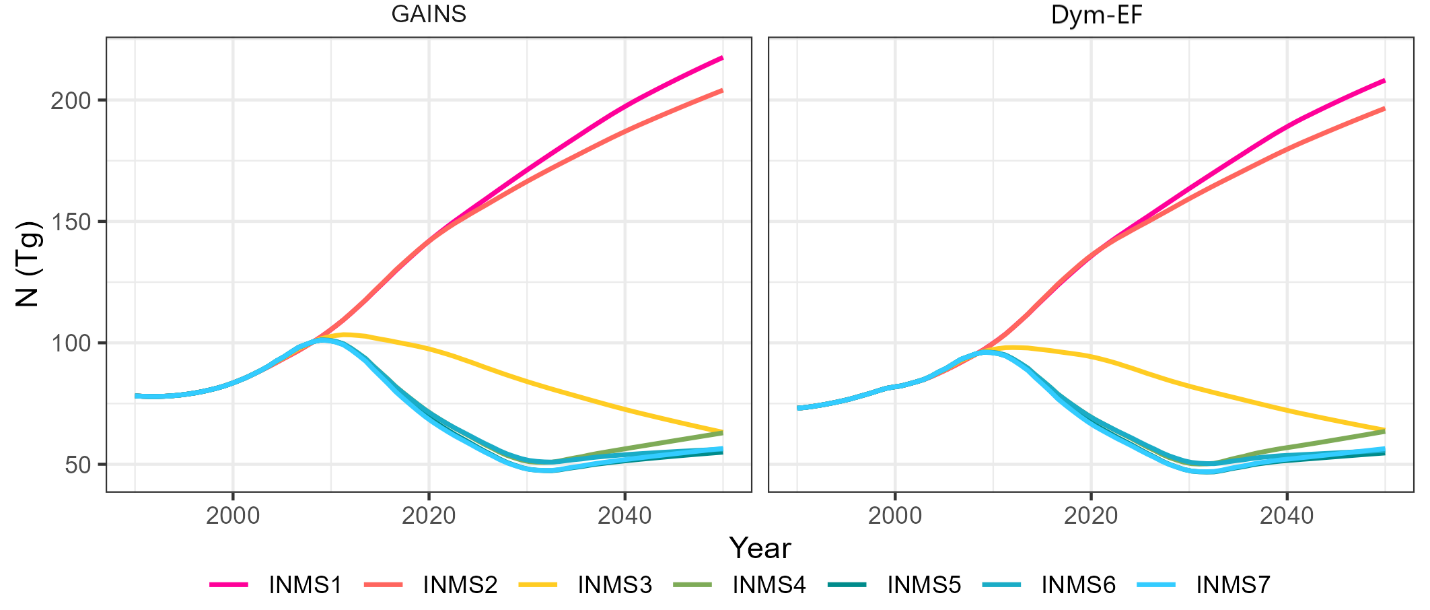


Figure S5 The projected global synthetic N fertilizer consumption under seven INMS scenarios at the region/country-scale used in GAINS (left) and at a half-degree resolution used in Dym-EF (right) during 1990-2050. INMS1 (Business-as-usual), INMS2 (Low N regulation), INMS3 (Medium N regulation), INMS4 (High N regulation), INMS5 (Best-case), INMS6 (Best-case Plus), and INMS7 (Bioenergy). Our data indicate that global synthetic N fertilizer input increased by 11.2% during 2011-2020.


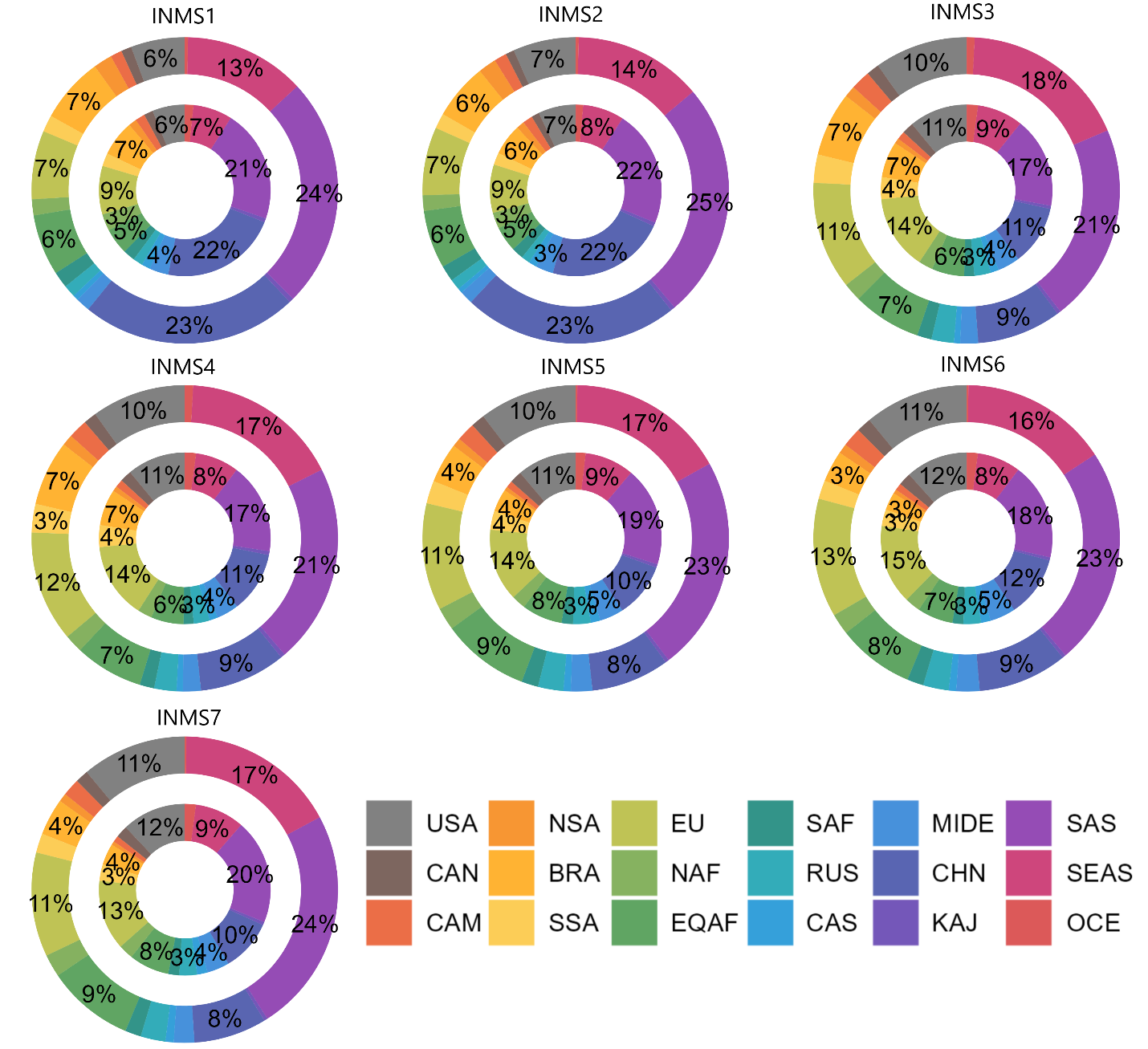


Figure S6 Share of global N₂O emission by each of the 18 sub-regions in 2050. The inner ring represents the N₂O emission share based on static EF and the outer circle represents the N₂O emission share based on Dynamic EF. The anti-clockwise color ring matches the region color shown in the legend.


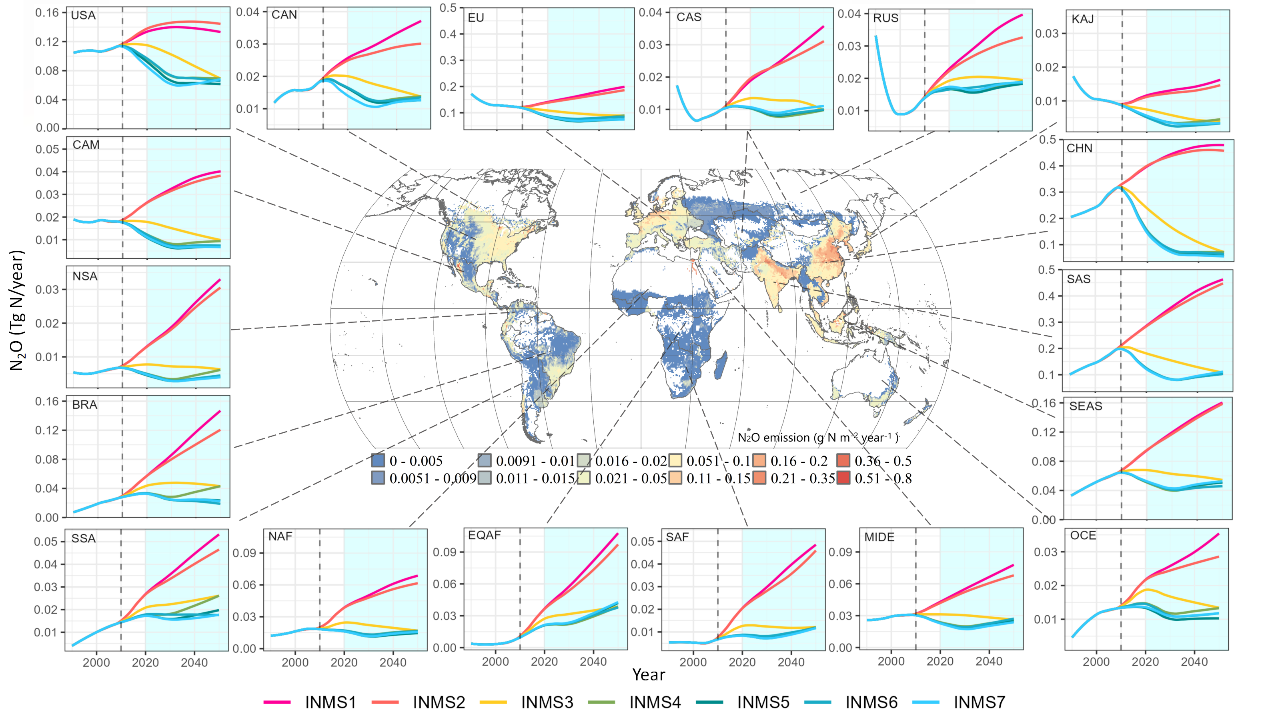


Figure S7 Projected N₂O emission across different subregions during 1990-2050 based on the static EFs. The white and blue-shaded time periods denote 1990-2020 and 2020-2050, respectively. The spatial map is N₂O emissions estimated by static EF in 2010. Key timeline markers include black and blue dashed vertical lines for the years 2010 and 2020, respectively. INMS1-7 are depicted as seven nitrogen management scenarios. BRA, Brazil; CAM, Central America; CAN, Canada; CAS, Central Asia; CHN, China; EQAF, Equatorial Africa; EU, Europe; KAJ, Korea and Japan; MIDE, Mideast; NAF, Northern Africa; NSA, Northern South America; OCE, Oceania; RUS, Russia; SAF, Southern Africa; SAS, South Asia; SEAS, Southeast Asia; SSA, Southwest South America; USA, The United States of America.


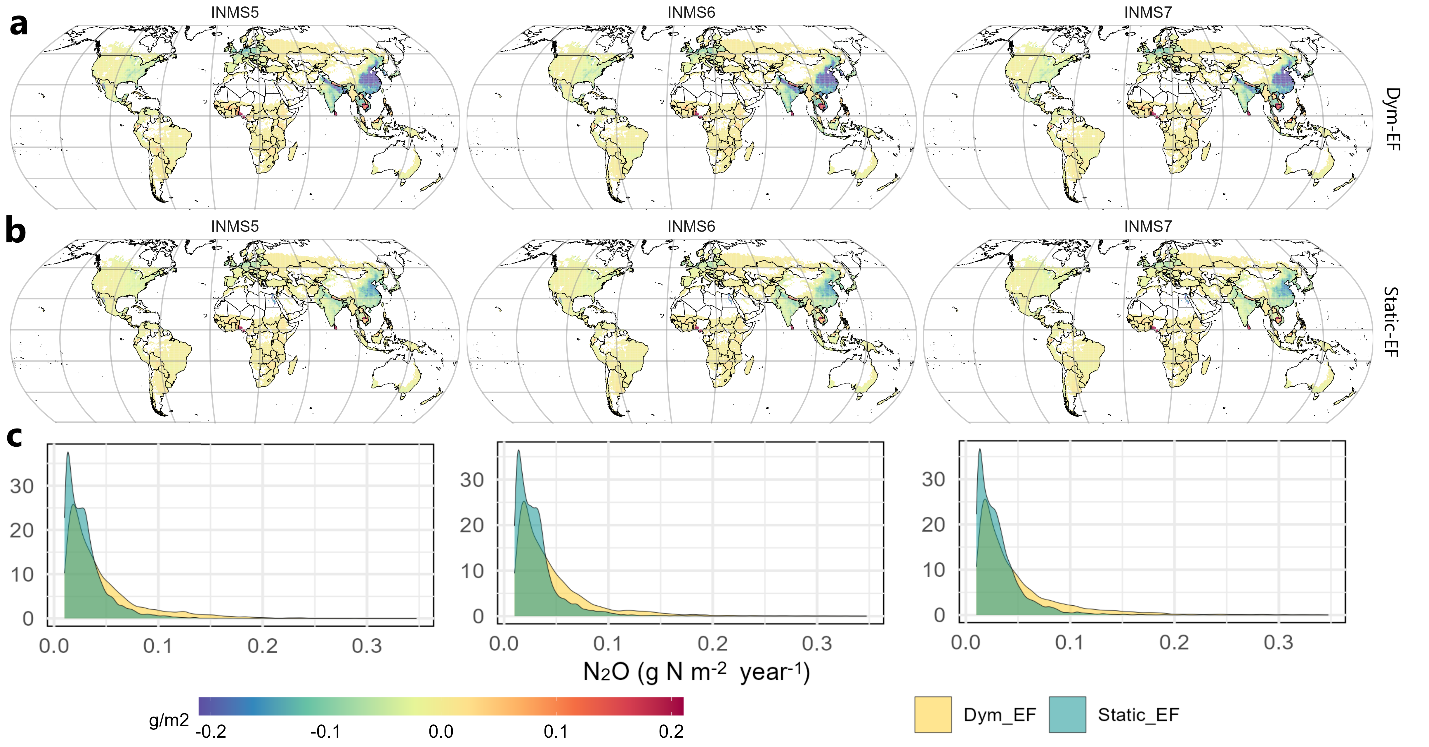


Figure S8 The projected N₂O emission changes at global scales in 2050 relative to the 2010 baseline under scenarios INMS5 (Best-case), INMS6 (Best-case “plus”), and INMS7 (Bioenergy). a, N_2_O projections using the Dym-EF. b, N_2_O projections using the static-EF. c, Comparison of probability density distributions of global N₂O Emissions in 2050 estimated by dynamic (Dym-EF, yellow) and static EF (Static-EF, blue) approaches for each scenario.


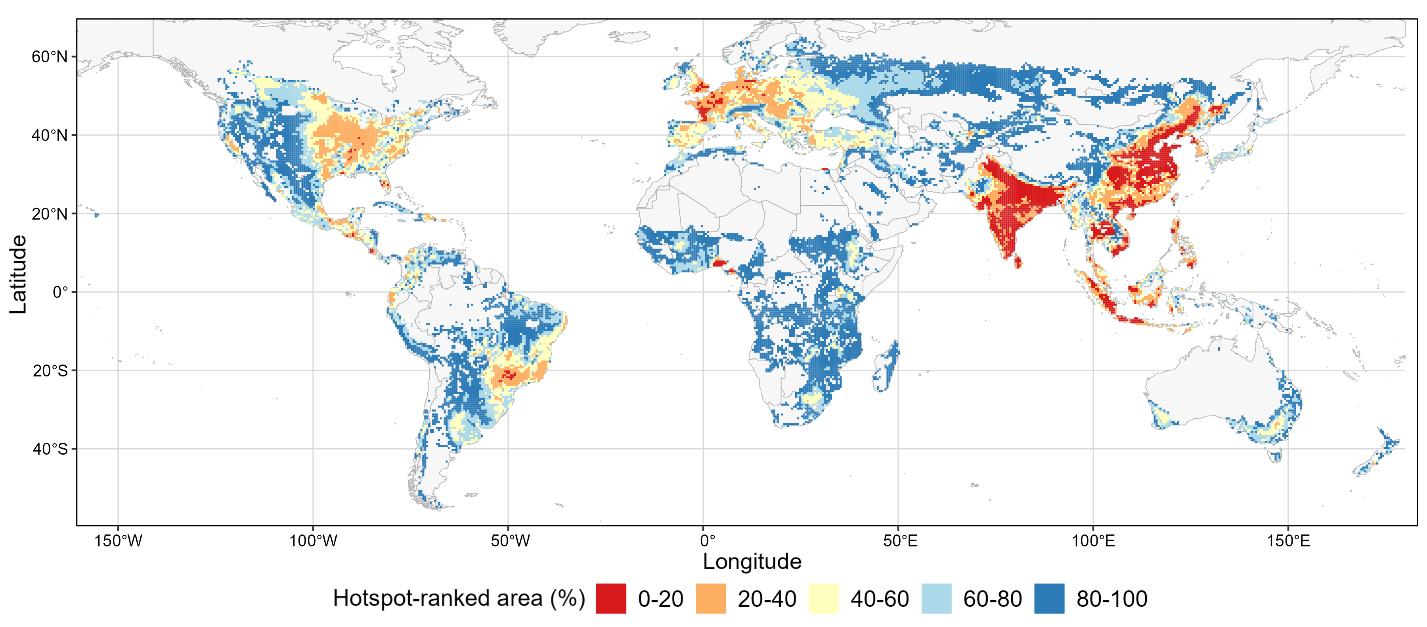


Figure S9 Spatial distribution of hotspot ranking based on historical N_2_O emission rates per square meter land in each half-degree grid cell, as estimated under current policies in 2020. This pattern was used for testing the N_2_O reduction potential of NI adoption in Fig 5a.

1. Tian, H. *et al.* The global N2O model intercomparison project. *Bull. Am. Meteorol. Soc.* **99**, 1231–1251 (2018).

2. Tian, H. *et al.* Global nitrous oxide budget (1980-2020). *Earth Syst. Sci. Data* **16**, 2543–2604 (2024).

3. Li, L. *et al.* Enhanced nitrous oxide emission factors due to climate change increase the mitigation challenge in the agricultural sector. *Glob. Chang. Biol.* **30**, 1–18 (2024).

4. Shcherbak, I., Millar, N. & Robertson, G. P. Global metaanalysis of the nonlinear response of soil nitrous oxide (N 2O) emissions to fertilizer nitrogen. *Proc. Natl. Acad. Sci. U. S. A.* **111**, 9199–9204 (2014).

5. Stehfest, E. & Bouwman, L. N2O and NO emission from agricultural fields and soils under natural vegetation: summarizing available measurement data and modeling of global annual emissions. *Nutr. Cycl. Agroecosystems* **74**, 207–228 (2006).

6. Harris, I., Osborn, T. J., Jones, P. & Lister, D. Version 4 of the CRU TS monthly high-resolution gridded multivariate climate dataset. *Sci. Data* **7**, 1–18 (2020).

7. Kim, D.-G., Hernandez-Ramirez, G. & Giltrap, D. Linear and nonlinear dependency of direct nitrous oxide emissions on fertilizer nitrogen input: A meta-analysis. *Agric. Ecosyst. Environ.* **168**, 53–65 (2013).

8. Strokov, A. S. & Potashnikov, V. Y. Environmental tradeoffs of agricultural growth in Russian regions and possible sustainable pathways for 2030. *Russ. J. Econ.* **8**, 60–80 (2022).

9. Laing, A. M., Eckard, R. J., Smith, A. P. & Grace, P. Twenty years of nitrous oxide emissions research in Australian agriculture: A review. *Agric. Ecosyst. Environ.* **356**, 108638 (2023).

10. Grace, P. *et al.* Revised emission factors for estimating direct nitrous oxide emissions from nitrogen inputs in Australia’s agricultural production systems: a meta-analysis. *Soil Res.* **62**, (2024).

11. Kanter, D. R. *et al.* A framework for nitrogen futures in the shared socioeconomic pathways. *Glob. Environ. Chang.* **61**, 102029 (2020).

12. Zhang, X. *et al.* Managing nitrogen for sustainable development. *Nature* **528**, 51–59 (2015).
